# Type I and III interferon responses restrict infection by tick-borne orthoflaviviruses through IFI6

**DOI:** 10.1128/jvi.00760-25

**Published:** 2026-04-14

**Authors:** Felix Streicher, Devin Kenney, Vincent Caval, Maxime Chazal, Sophie-Marie Aicher, Ségolène Gracias, Scott Adams, Mao Matsuo, Ferdinand Roesch, Florian Douam, Nolwenn Jouvenet

**Affiliations:** 1 Virus sensing and signaling Unit, Institut Pasteur, Université Paris Cité, CNRS UMR 3569 555089, Paris, France; 2 Department of Virology, Immunology and Microbiology, Boston University School of Medicine 12259 , Boston, Massachusetts, USA; 3 National Emerging Infectious Diseases Laboratories, Boston University 1846, Boston, Massachusetts, USA; 4 UMR 1282 Infectiologie et Santé Publique, INRAE Centre Val de Loire 56586, Nouzilly, France; The Ohio State University, Columbus, Ohio, USA

**Keywords:** RNA virus, flaviviruses, innate immunity, interferons, tick-borne pathogens, animal models, host-pathogen interactions

## Abstract

Tick-borne orthoflaviviruses (TBOVs) are a growing global health concern. Several members of this viral group cause fatal disease in humans with increasing case numbers throughout the last few decades. The innate immune response, especially interferon (IFN)-dependent signaling, is an essential part of the vertebrate defense system that counteracts infection with TBOVs and other viruses. Although they activate the same signaling cascade, IFNs belonging to the type I and III families trigger differing gene expression patterns. Which genes the two IFN families induce to restrict infection with TBOVs remains poorly characterized. Here, we show that type I and III IFNs are both capable of restricting TBOV infection of human cell lines in a cell type-specific manner. Infection of C57BL/6J mice with knockouts for either IFN type I or type I and III receptors further underscored the critical role of IFN signaling in controlling TBOV replication *in vivo*. To assess the contribution of single genes to controlling TBOV infection in human cells, we used a CRISPR/Cas9-KO-based screening approach. This strategy identified IFI6 as a central player for IFN type I- and III-driven responses against TBOVs. We further defined IFI6 as an ER-resident protein that restricts TBOV replication at a post-entry step. Our work thus opens new perspectives for targeting weak points in the life cycle of TBOVs and other orthoflaviviruses, potentially paving the way for the development of new antiviral therapeutics.

**IMPORTANCE:**

Tick-borne orthoflaviviruses (TBOVs) are spreading in various parts of the world, like Europe, Asia, and North America, making it essential to understand how they cause disease and how the immune system responds to infection. In vertebrates, the interferon (IFN) response is a key early defense against viruses, triggering the expression of numerous IFN-stimulated genes (ISGs) with antiviral activities. Using mouse models, we demonstrated the central role of IFNs in controlling TBOV replication. To explore this further, we screened for the activity of about 2,000 individual ISGs against tick-borne encephalitis virus (TBEV) in human cells and identified IFI6 as a potent antiviral factor. Through functional studies, virological assays, biochemical analyses, and microscopic approaches, we confirmed that IFI6 limits the replication of TBOVs. These findings enhance our understanding of innate immunity against TBOV infections.

## INTRODUCTION

Tick-borne orthoflaviviruses (TBOVs) are enveloped viruses with a 10–11 kb long, single-stranded, positive-sense RNA genome encoding three structural (capsid [C], membrane [M], and envelope [E]) and seven non-structural (NS1, NS2A, NS2B, NS3, NS4A, NS4B, and NS5) proteins (1). Much like their more extensively studied mosquito-borne relatives, such as Zika virus (ZIKV), dengue virus (DENV), or yellow fever virus (YFV), TBOVs pose a major threat to global health (1–4). Several TBOVs cause severe diseases in humans and can be divided into viruses that primarily induce hemorrhagic or neuronal pathology. Members of the latter, such as tick-borne encephalitis virus (TBEV), Powassan virus (POWV), or louping-ill virus (LIV), infect the human central nervous system (CNS) and cause long-term neurological damage or death (3, 5–7). They are transmitted by a variety of mostly hard-bodied ticks of predominantly *Ixodes* and *Dermacentor spp*. that are distributed worldwide with a geographical preference for the temperate zone of the northern hemisphere (2, 3). Although most human infections occur through tick bites, numerous cases of TBOV infection after ingestion of unpasteurized milk stemming from infected livestock such as sheep and goats have been reported in humans (8, 9). As of 2025, TBEV, the prototypical neuropathogenic TBOV that is responsible for the most severe infections in humans, is the only TBOV against which a vaccine has been approved for use in humans. Despite this, the incidence of TBEV-induced cases of severe disease has steeply increased throughout the last decades (10–12). This is not only linked to a constant expansion of the habitats of the vector tick species and low vaccination rates but also results from the lack of antiviral treatment options against TBOVs (7, 13, 14). To counteract the continuous rise in the number of patients suffering from severe disease caused by these emerging pathogens, alternative strategies against TBOVs need to be explored.

The first line of defense against pathogenic intruders in eukaryotes is the innate immune system (15). The vertebrate innate immune system has evolved to rapidly control virus replication by inducing the expression of antiviral cytokines like type I and III interferons (IFNs). Upon secretion by infected cells, IFN type I (IFNα, IFNβ) and type III (IFNλ, also known as IL29, IL28A, and IL28B) bind to their heterodimeric receptors, IFNAR1/IFNAR2 and IFNλR1/IL-10R2, respectively, and activate the canonical JAK/STAT pathway in infected and surrounding cells (16). Activation of this pathway results in the expression of a plethora of IFN-stimulated genes (ISGs) that concertedly establish an antiviral state in the cells. ISGs comprise a core set of genes that, upon stimulation, are induced at high levels in all cell types across mammalian species (17). Certain ISGs directly block the viral life cycle by targeting specific stages of virus replication, including entry into host cells, protein translation, replication, or assembly of new viral particles (18). While some ISGs display virus-specific (or viral family-specific) antiviral activity, others exhibit broad-spectrum antiviral functions. Furthermore, ISGs are also involved in the regulation of IFN signaling and thus are key for facilitating the return to cellular homeostasis. However, the contribution of most ISGs to the modulation of viral infection and replication remains poorly characterized (18).

Despite both activating the JAK/STAT pathway, IFN-I and IFN-III stimulate ISGs with different kinetics and potency, and in a cell type-dependent manner (19–22). These differences are likely connected to the abundance of type I or III receptor complexes expressed at the surface of different cell types (21). While type I IFNs and their receptors are ubiquitously expressed and robust inducers of the antiviral state in most cell types, type III IFN receptor complexes are expressed in a limited set of cell types, mainly in cells that compose mucosal surface tissues, like the lung or the gut (23–26). The importance of IFN-I signaling in controlling TBOV infections has been previously established in several distinct human cell types *in vitro* and in various murine models *in vivo* (6, 27–36). In contrast, the antiviral potential of type III IFNs against TBOVs remains unclear. The only study investigating this aspect reported no anti-TBEV activity of IFN-III in human medulloblastoma cells (30). In addition, although the antiviral activities of IFN-I against TBOVs are documented, their effectors are poorly characterized.

Aiming to clarify this, we investigated the impact of IFN-I and -III on TBOV replication *in vitro* and *in vivo* and performed the first ISG-centric CRISPR/Cas9 knock-out genetic screen for TBOV to identify which IFN-induced effector(s) contribute(s) to the anti-TBOV state in human cells.

## RESULTS

### Human cell lines derived from tissues with physiological relevance to TBOV pathogenesis are susceptible to infection with two TBOVs

**Fig 1:**
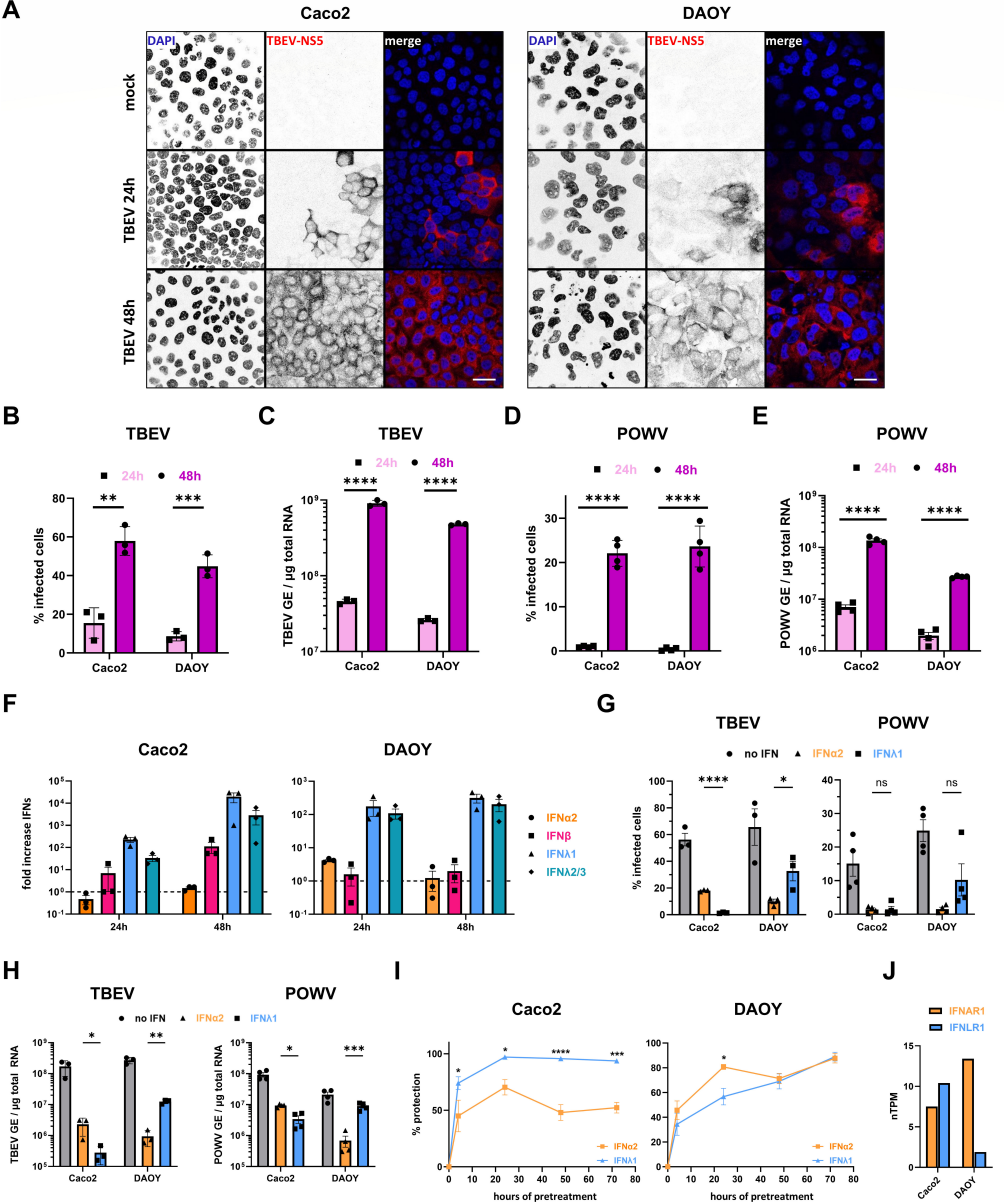
Type I and III IFNs protect human cell lines with physiological relevance against infection with TBOVs in a cell type-specific manner. (**A**) Confocal microscopy analysis of human colorectal adenocarcinoma (CaCO_2_) or medulloblastoma cells (DAOY) infected with TBEV-Hypr at an MOI of 0.1. Twenty-four and 48 h post-infection, the cells were fixed, stained for TBEV-NS5 protein (red), and their nuclei were stained with DAPI (blue). Scale bar: 30 µm. Images are representative of two independent experiments. (**B–E**) CaCO_2_ or DAOY cells were infected with either TBEV-Hypr (MOI 0.1) or POWV-SPO (MOI 5) and monitored for percentage of infected cells via cytometric analysis after staining for orthoflaviviral E protein (**B and D**) or for relative amounts of viral RNA, expressed as genome equivalents (GE), via RT-qPCR analysis (**C and E**) at 24 and 48 hpi. (**F**) RT-qPCR analysis of mRNA levels of different interferons in Caco2 or DAOY cells infected with TBEV-Hypr at an MOI of 1 for 24 or 48 h. (**G and H**) Analysis of Caco2 and DAOY cells treated with either IFNα2 (0.25 ng/mL, orange) or IFNλ1 (20 ng/mL, blue) for 16 h, or left untreated and then infected with either TBEV-Hypr (MOI 1, 24 h) or POWV-LB (MOI 5, 48 h). Cells were either stained for orthoflaviviral envelope protein and subjected to flow cytometric analysis to identify the percentage of infected cells (**G**), or the relative amounts of viral RNA, expressed as genome equivalents (GE), were determined via RT-qPCR analysis (**H**). (**I**) Caco2 or DAOY cells were treated with IFNα2 (0.25 ng/mL, orange) or IFNλ1 (20 ng/mL, blue) for 4, 24, 48, or 72 h and infected with TBEV-Hypr (MOI 1) for 24 h before being stained for orthoflaviviral envelope protein and subjected to cytometry-based analysis for infection levels. Those were then compared to the equivalent infection rates of untreated cells to identify the protective potential of the IFNs. (**J**) The abundance of IFNAR1 and IFNLR1 in cells of the Caco2 and DAOY cell lines, as archived in the human protein atlas database (https://www.proteinatlas.org/ENSG00000142166-IFNAR1/cell+line; https://www.proteinatlas.org/ENSG00000185436-IFNLR1/cell+line) displayed as nTPM (transcripts per million). Data are from three (B, C, F, G [TBEV], H [TBEV], and I) or two (D, E, G [POWV] and H [POWV]) independent experiments ± SEM. ns = non-significant, *P < 0.05, **P < 0.01, ***P < 0.001, ****P < 0.0001 by unpaired (**B, D, G**), lognormal (**C, E, H**) *t*-test with Holm-Sidak post-test or two-way ANOVA with Sidak post-test (**I**).

The transmission of TBOVs to humans can occur either through tick bite or ingestion of unpasteurized milk from infected animals (3, 7, 9). The latter marks cells of the intestinal epithelium as potential first targets of TBOV infections following oral transmission, which has been sparsely investigated to date (37). Independent of the transmission route, viral replication in neurons constitutes an essential part of the pathogenesis of neurotropic TBOVs (38). Thus, we selected human colorectal adenocarcinoma (Caco2) and medulloblastoma (DAOY) cells as human cell lines to study TBOV replication, since both were previously reported to be susceptible to TBEV (30, 37). Caco2 and DAOY cells were infected with TBEV (strain Hypr) at a multiplicity of infection (MOI) of 0.1. Of note, since infectivity was assessed by titration on Vero cells, MOI refers to Vero cell MOI. Viral replication was monitored over time by analyzing the expression of the viral protein NS5 through immunofluorescence imaging at 24 and 48 h post-infection (hpi) (Fig. 1A). Both cell lines were susceptible to TBEV, with an increase in NS5-positive cells over time (Fig. 1A). In line with this, flow cytometric analysis using pan-orthoflavivirus anti-E antibody revealed that while about 15% of Caco2 and 10% of DAOY cells were infected at 24 hpi, this increased to 55% and 45%, respectively, at 48 hpi (Fig. 1B). Consistently, assessment of viral replication by RT-qPCR analysis showed a significant increase in viral RNA over time (Fig. 1C), further indicating robust TBEV replication in both cell lines. To assess whether these two cell lines were susceptible to other members of the TBOV group, they were infected with POWV (strain SPO) at an MOI of 5. About 20%–25% of Caco2 and DAOY cells were positive for viral E proteins at 48 hpi (Fig. 1D). Viral RNA yields significantly increased over time (Fig. 1E), further indicating viral replication. However, viral RNA abundances were about 1 log lower in POWV-infected cells than in TBEV-infected cells (Fig. 1C and E). In sum, despite disparities, Caco2 and DAOY cells were susceptible to TBEV and POWV. This validates these cell lines as useful models approximating tissues with physiological relevance to TBOV pathogenesis.

### Type I and III IFNs protect human cell lines against infection with TBOVs in a cell type-specific manner

Type I IFNs were previously shown to have a protective effect against several TBOVs *in vitro* (6, 27, 30, 34). In contrast, the antiviral potential of the type III IFN family against TBOVs remains poorly characterized. We examined mRNA abundance of four IFN genes (*IFNA2*, *IFNB*, *IFNL1,* and *IFNL2/3*) in TBEV-infected cells at 24 and 48 hpi. Type III IFN upregulation surpassed type I IFN upregulation in TBEV-infected Caco2 and DAOY cells at both time points (Fig. 1F), potentially indicating an important role for type III IFNs in the antiviral response against TBOVs. We then evaluated the effect of IFN treatments on TBEV replication in both cell lines. We chose the concentrations of 20 ng/mL for IFNλ1 and 0.25 ng/mL for IFNα2 since they led to over 75% reduction in the number of E-positive cells, in Caco2 and DAOY cells, respectively (Fig. 1G). These concentrations of IFN-I and -III approximate physiological levels reported in TBEV-infected patients, where serum IFN-I levels ranged from 2.5 to 350 pg/mL, and IFN-III levels reached up to 1 ng/mL in cerebrospinal fluid (39, 40). Additionally, these concentrations align with the levels of IFNα detected in the serum of C57BL/6J mice infected for 2 days with POWV, either subcutaneously or orally (Fig. S1). Our results show that both IFN treatments significantly inhibited TBEV replication in Caco-2 and DAOY cells (Fig. 1G and H). Similarly, POWV replication was significantly restricted in Caco-2 and DAOY cells treated with IFN (Fig. 1G and H). We also investigated the protective effect of IFNα2 and IFNλ1 against TBEV infection after varying periods of pre-treatment. Caco2 cells were better protected by IFNλ1 than IFNα2 pre-treatment against infection with TBEV, independent of the timing (Fig. 1I). At earlier time points post-treatment, DAOY cells were better protected by IFNα2 than by IFNλ1 (Fig. 1I). However, after extended incubation times, the two treatments triggered the same protective state (Fig. 1I). To assess whether the varying degrees of protection were linked to the expression of the IFN receptor complexes in the two cell types, transcriptomic data from the human protein atlas were exploited (41). While Caco2 cells present a higher relative expression of *IFNLR1*, as compared to DAOY cells, DAOY cells express comparatively more *IFNAR1* than Caco2 cells (Fig. 1J). This suggests that the abundance of receptors indeed influences the responsiveness to different IFN types and subsequently, the ability to control viral replication. Together, these data show that both type I and III IFNs mount an effective antiviral state against TBEV and POWV in Caco2 and DAOY cells with cell type-specific differences in potency of the antiviral response, which is likely related to the expression level of IFN receptors.

### Type I IFN responses protect C57BL/6J mice against early death and rapid viral dissemination

**Fig 2:**
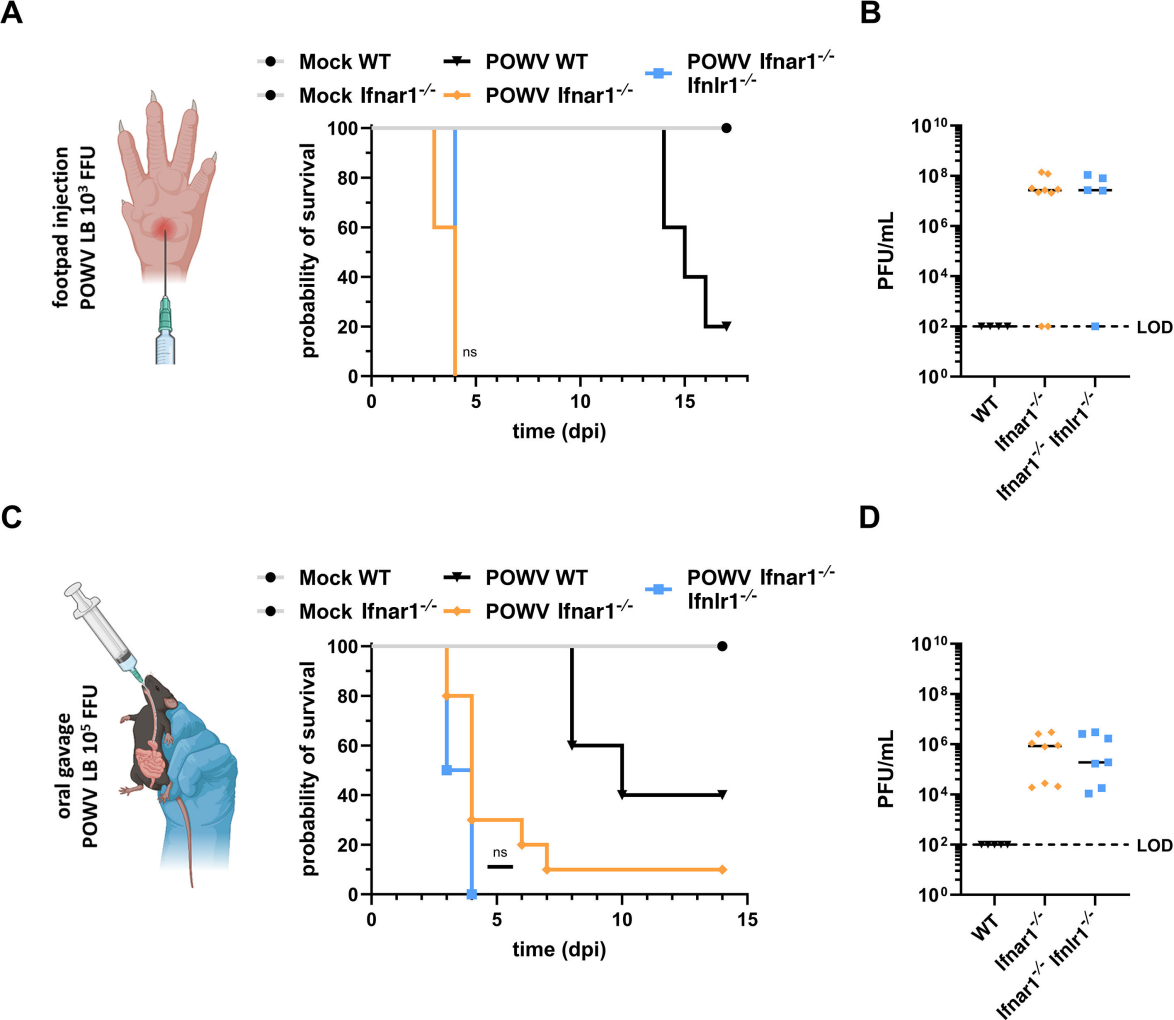
*Ifnar1^-/-^* and *Ifnar1^-/-^ Ifnlr1^-/-^* mice are hypersusceptible to POWV infection. (**A**) Survival of WT (black), *Ifnar1^-/-^* (orange), and *Ifnar1^-/-^ Ifnlr1^-/-^* (blue) C57BL/6J mice (9–20 weeks of age, 5–10 per group) following subcutaneous POWV-LB infection with 10^3^ FFU or WT and *Ifnar1^-/-^* (gray, 2–3 per group) after administration of PBS as a control. (**B**) Titration of infectious viral particles in the sera of WT (black), *Ifnar1^-/-^* (orange), and *Ifnar1^-/-^ Ifnlr1^-/-^* (blue) C57BL/6J mice subcutaneously infected with 10^3^ FFU of POWV-LB (5–9 per group), as determined 2 dpi via plaque assay. (**C**) Survival of WT (black), *Ifnar1^-/-^* (orange), and *Ifnar1^-/-^ Ifnlr1^-/-^* (blue) C57BL/6J mice (5–9 per group) following POWV-LB infection with 10^5^ FFU through oral gavage or WT and *Ifnar1^-/-^* (gray, 2–3 per group) after administration of PBS as a control. (**D**) Titration of infectious viral particles in the sera of WT (black), *Ifnar1^-/-^* (orange), and *Ifnar1^-/-^ Ifnlr1^-/-^* (blue) C57BL/6J mice orally infected with 10^5^ FFU of POWV-LB (4–8 per group), as determined 2 dpi via plaque assay. Limits of detection (LOD) are as indicated. Statistical significance (ns = non-significant) was assessed by log-rank (Mantel-Cox) test.

Several studies have demonstrated a critical role of type I IFN signaling in preventing swift and widespread TBOV infection in mice, as well as rapid lethality (29, 37, 42). However, the impact of type I IFN signaling during POWV infection *in vivo*, particularly across multiple inoculation routes, has not been comprehensively investigated. Furthermore, it remains unknown whether type III IFNs exert any protective effects against TBOV infection, at the clinical and/or virological levels, when type I IFN responses are compromised. C57BL/6J mice genotyped as either wild-type (WT), *Ifnar1^-/-^*, or *Ifnar1^-/-^ Ifnlr1^-/-^* were first infected with POWV (strain LB, 10^3^ FFU) via subcutaneous injection into the footpad (Fig. 2A and B). The survival rates of the infected mice were monitored for a 17-day period (Fig. 2A), and viral titers in the sera were measured through plaque assay at 2 dpi (Fig. 2B). PBS-injected control mice survived the complete experiment without developing any clinical signs, independent of their genotype (Fig. 2A). WT C57BL/6J mice exhibited an 80% mortality rate after 16 days of infection (Fig. 2A). All *Ifnar1^-/-^* mice rapidly succumbed to infection, some as early as 3 dpi and at the latest at 4 dpi (Fig. 2A). Similarly, *Ifnar1^-/-^ Ifnlr1^-/-^* mice died 4 days after injection of POWV (Fig. 2A). No infectious POWV particles were detected in the sera of WT C57BL/6J mice at 2 days post-footpad injection, in line with previous findings (43). Depletion of type I or type I and III IFN signaling resulted in high serum viral titers at 2 dpi (Fig. 2B), albeit no differences were observed between the two murine genotypes. Therefore, following subcutaneous infection, both *Ifnar1^-/-^* and *Ifnar1^-/-^ Ifnlr1^-/-^* mice exhibited similar hypersusceptibility to POWV infection and fatal disease. Our findings suggest that IFN-I signaling restricts early, widespread POWV dissemination and disease in mice, whereas type-III IFNs appear to exert no protective functions in this context. Due to the rapid lethality associated with the loss of IFN-I signaling, it is difficult to definitively assess whether IFN-III might have an additive or synergistic impact on disease severity during POWV infection.

Our *in vitro* experiments using the Caco2 cell line suggest that epithelial barrier cells may respond more robustly to IFN-III than IFN-I (Fig. 1H). To test whether this observation translates into a substantial role for type III IFN-signaling in limiting early viral dissemination and/or disease in mice lacking type I IFN signaling, we infected WT, *Ifnar1^-/-^*, or *Ifnar1^-/-^ Ifnlr1^-/-^* C57BL/6J mice with 10^5^ infectious particles of POWV administered via oral gavage (Fig. 2C). Mice were either monitored for survival over 14 days (Fig. 2C) or sacrificed at 2 dpi, and their sera analyzed for viral titers (Fig. 2D). Mock-treated mice remained unaffected throughout the monitored period, whereas 60% of WT mice infected succumbed to POWV infection within 10 days (Fig. 2D). All *Ifnar1^-/-^ Ifnlr1^-/-^* mice died within the first 4 days post-infection, while 30% of *Ifnar1^-/-^* mice were still alive at this time point (Fig. 2C). The *Ifnar1^-/-^* mice that were still alive at 4 dpi, except for one that survived the entire monitoring period, died within the following 3 days. This delay in lethality, albeit non-significant, may suggest a potential role for IFN-III in limiting early POWV disease when type I IFN signaling is absent. However, viremia levels in the sera at 2 dpi were comparable between *Ifnar1^-/-^* and *Ifnar1^-/-^ Ifnlr1^-/-^* mice, although the detected titers were 2–3 logs lower in orally infected mice compared to those infected subcutaneously at the same time point (Fig. 2B and D).

Together, these results suggest that type I IFN is the primary mediator of the antiviral response against POWV *in vivo* and that type III IFN signaling does not appear critical for preventing rapid viremia or accelerated death in C57BL/6J mice, irrespective of the inoculation route.

### Type I IFN responses prevent POWV dissemination across a wide range of tissues in *Ifnar1^-/-^* C57BL/6J mice in an inoculation route-dependent manner

**Fig 3:**
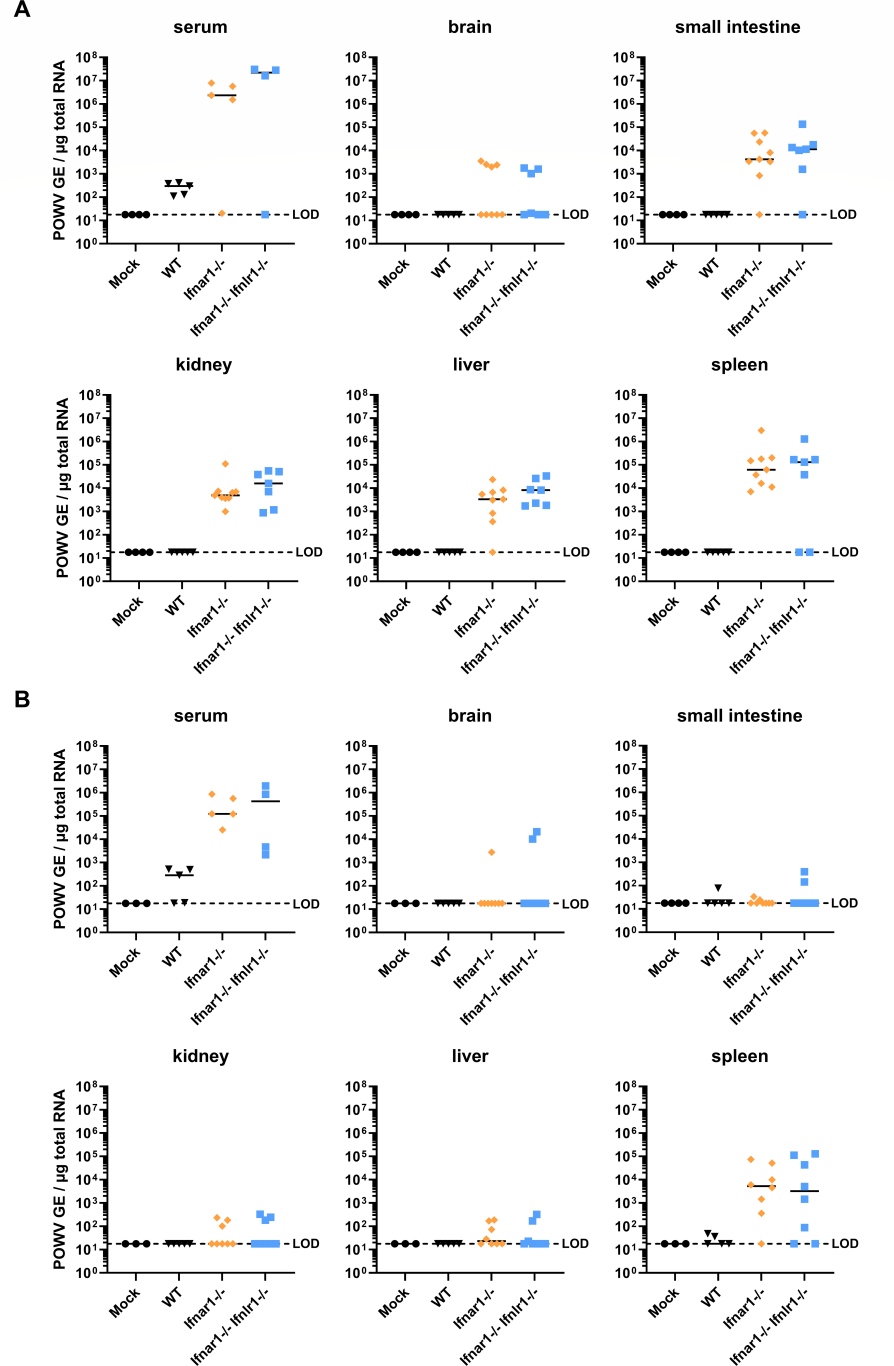
POWV disseminates into several peripheral tissues in *Ifnar1^-/-^* and *Ifnar1^-/-^ Ifnlr1^-/-^* mice. (**A**) Viral loads within serum, brain, small intestine, kidney, liver, and spleen tissue of WT (black), *Ifnar1^-/-^* (orange), and *Ifnar1^-/-^ Ifnlr^-/-^* (blue) C57BL/6J mice (9–20 weeks of age, 4–9 per group) subcutaneously infected with 10^3^ FFU of POWV-LB, or (**B**) of WT (black), *Ifnar1^-/-^* (orange), and *Ifnar1^-/-^ Ifnlr1^-/-^* (blue) C57BL/6J mice (3–8 per group) infected with 10^5^ FFU of POWV-LB through oral gavage at 2 dpi. Viral positive-strand RNA copies were quantified by RT-qPCR and expressed as genome equivalents (GE) per microgram total RNA. Limits of detection (LOD) are as indicated.

To characterize the effect of IFN signaling on POWV dissemination and tropism, WT, *Ifnar1^-/-^*, or *Ifnar1^-/-^ Ifnlr1^-/-^* C57BL/6J mice were infected either by subcutaneous injection into the footpad (10^3^ FFU, Fig. 3A) or via oral gavage (10^5^ FFU, Fig. 3B). Viral RNA abundance in serum and individual tissues was quantified by RT-qPCR analysis at 2 dpi. All tested tissues from mock-treated and WT mice subcutaneously infected with POWV were negative for viral RNA (Fig. 3A), indicating that POWV replicated less efficiently in WT mice than in mice lacking IFN receptors. By contrast, most or all *Ifnar1^-/-^* and *Ifnar1^-/-^ Ifnlr1^-/-^* mice exhibited viral RNA in the kidney, liver, spleen, and small intestine (Fig. 3A). The highest viral RNA levels were observed in the spleen, suggesting that this tissue is particularly dependent on IFN signaling to limit viral dissemination. Only 44% and 42% of *Ifnar1^-/-^* and *Ifnar1^-/-^ Ifnlr1^-/-^* mice, respectively, showed viral RNA in the brain, highlighting that early death in mice with compromised type I IFN signaling does not necessarily associate with neuroinvasion at 2 dpi (Fig. 3A). No significant differences in viral RNA load were observed between the two genotypes (Fig. 3A), indicating that deficiency in type I IFN signaling drives widespread viral dissemination and broad tissue tropism in subcutaneously inoculated C57BL/6J mice. Oral infection of WT, *Ifnar1^-/-^*, or *Ifnar1^-/-^ Ifnlr1^-/-^* C57BL/6J mice with POWV (10^5^ FFU) resulted in reduced viral dissemination across tissues at 2 dpi, despite a 100-fold higher infectious dose compared to the subcutaneous route. Most or all *Ifnar1^-/-^* and *Ifnar1^-/-^ Ifnlr1^-/-^* mice showed the presence of viral RNA in the serum and the spleen (Fig. 3B), while only a limited proportion (12%–37% and 25%–37%, respectively) displayed viral RNA in the small intestine, kidney, liver, and brain. No significant differences in viral yield were observed between *Ifnar1^-/-^* and *Ifnar1^-/-^ Ifnlr1^-/-^* mice. Collectively, our findings show that type I IFNs restrict rapid dissemination into peripheral tissues, particularly in the spleen. However, the oral route seems less effective in driving systemic dissemination upon disruption of IFN responses than the footpad route, despite a similar lethal phenotype (both temporally and clinically). Type III IFN signaling does not appear to play a substantial role in limiting viral dissemination in this context, regardless of the inoculation route.

### A CRISPR-KO screen identifies IFI6 as the central effector of the IFN type I- and III-driven antiviral responses against TBEV

**Fig 4:**
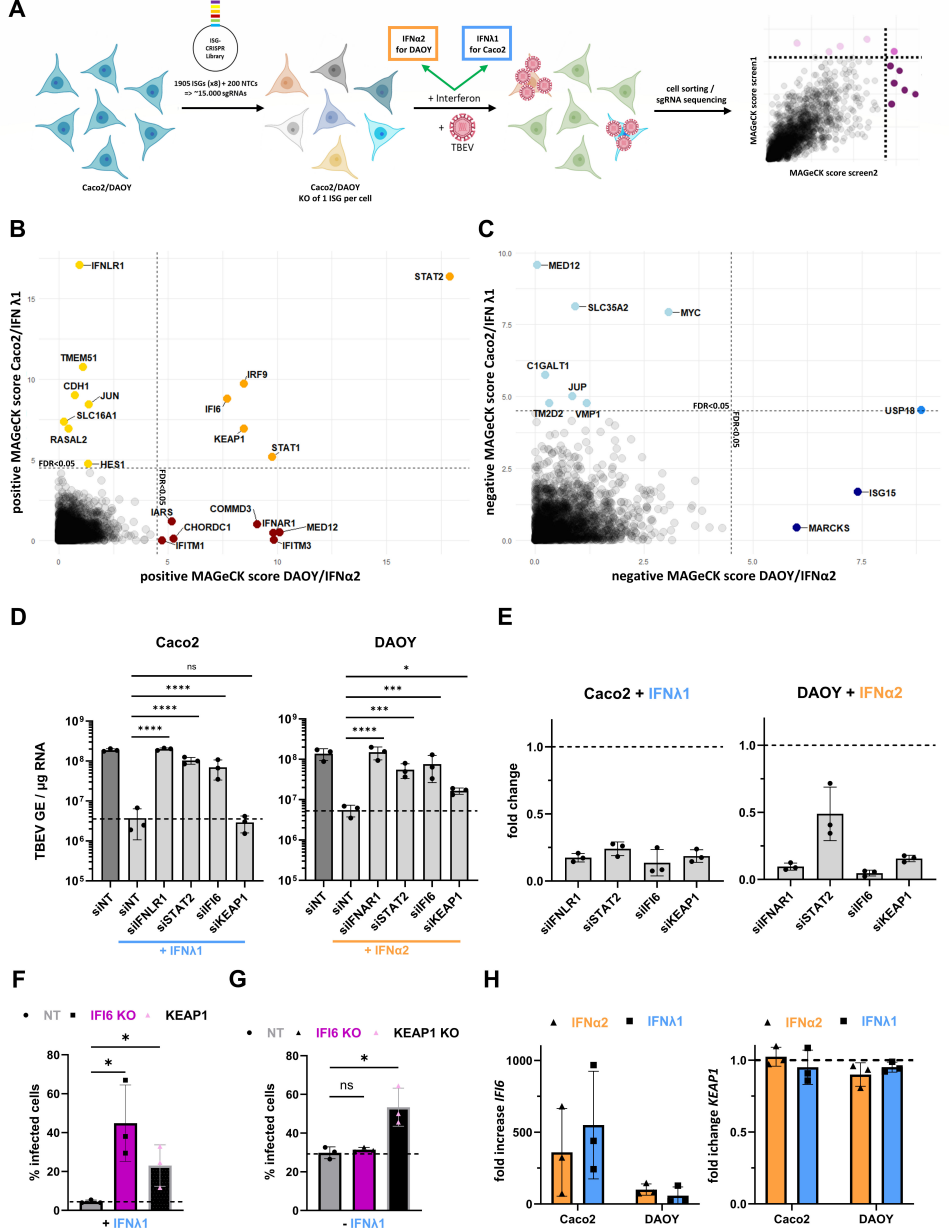
A CRISPR-KO screen identifies IFI6 as the central effector of the IFN type I and III-driven antiviral responses against TBEV. (**A**) Schematic flow chart of the CRISPR-KO screen setup. (**B**) Antiviral and (**C**) proviral hits of the screens as identified through analyses of the resulting NGS data with the MAGeCK package. The cutoff point for screen hits is an FDR of 0.05. (**D**) Caco2 or DAOY cells were issued to siRNA-based knockdown of antiviral screen hits, or transfected with non-targeting siRNA (siNT), treated with IFNλ1 (Caco2, 16 h, 0.1 ng/mL) or IFNα2 (DAOY, 16 h, 10 ng/mL), infected with TBEV-Hypr (MOI 1, 24 h) and examined for viral genomic copy numbers via RT-qPCR. (**E**) Efficiency of knockdown of expression of target genes in the same IFN-treated samples as in panel D compared to cells transfected with siNT, as assessed by RT-qPCR analysis. (**F**) Caco2 cells with KO of *IFI6* or *KEAP1* or control cells (NT) were treated with IFNλ1 (16 h, 20 ng/mL) and infected with TBEV-Hypr at MOI 0.1 for 24 h. The percentage of infected cells was determined by flow cytometry after staining for orthoflaviviral envelope protein. (**G**) Same setup as panel F without IFN treatment. (**H**) Caco2 or DAOY cells were treated with either IFNα2 (1.25 ng/mL, orange) or IFNλ1 (100 ng/mL, blue) for 16 h and analyzed for mRNA expression levels of *IFI6* and *KEAP1* via RT-qPCR. Data (**D–H**) are from three independent experiments ± SEM. ns = non-significant, *P < 0.05, ***P < 0.001, ****P < 0.0001 by log-normal ordinary one-way ANOVA with Dunnett post-test (**D**), or unpaired, two-tailed *t*-test (**F and G**). Panel A was created using Biorender.com.

To identify the IFN effectors involved in the antiviral response against TBOVs, we established a CRISPR/Cas9 knockout (KO) screening approach targeting 1,905 genes previously identified as ISGs across various human cell lines and primary immune cells (Fig. 4A) (44). Although our experimental framework did not detect a contribution of type III IFN signaling in restricting fulminant POWV disease, we conducted the screen in Caco-2 cells treated with IFNλ1 and DAOY cells treated with IFNα2, as these treatments exhibited the most protective effects in our *in vitro* models (Fig. 1G through J). Cells transduced with the sgRNA library were selected by puromycin treatment, then treated with IFN, and finally infected with TBEV at an MOI of 1. Infected cells were sorted using antibodies binding to orthoflaviviral E protein, and DNA was extracted from infected and non-infected control cells to identify integrated sgRNA sequences in each cell fraction (Fig. 4A). Next-generation sequencing coupled to MAGeCK-based statistical analyses (45) led to the identification of sgRNAs targeting 19 genes significantly enriched in infected cells, representing potential antiviral factors (Fig. 4B). Comparison of antiviral hits in the two models revealed five genes that may be essential for both type I and III antiviral signaling against TBEV (Fig. 4B). Three of these genes are key members of the JAK/STAT pathway and thus well-known broadly acting IFN-I/III effectors: *STAT1*, *STAT2,* and *IRF9* (15, 16). *IFI6* and *KEAP1* were the other two antiviral candidates common to the two stimuli. Antiviral hits that were distinct for the two models included the receptors of the respective IFN type used for the two screens, namely *IFNLR1* for the type III/Caco2 screen and *IFNAR1* for the type I/DAOY screen (Fig. 4B), further validating our experimental approach. With *IFITM1* and *IFITM3*, ISGs that were previously observed to demonstrate antiviral effects against TBOVs were also detected here (41). Ten of the antiviral hits, such as *TMEM51* and *COMMD3,* have not been associated with antiviral functions yet. We also identified 10 genes that were significantly depleted in infected cells, representing potential proviral ISGs (Fig. 4C). These proviral hits included genes that are broad-spectrum facilitators of viral infection, such as *ISG15* and *USP18*, which negatively regulate the JAK/STAT pathway (46, 47). Several genes with unknown functions in the context of viral infection, such as *SLC35A2* and *MARCKS,* were also identified (Fig. 4C).

We focused on *IFI6* and *KEAP1,* which were the two antiviral hits that were common to the two screens. To further investigate their antiviral potential, their expression was knocked down using pools of four siRNAs. Negative controls were non-targeting siRNAs. Pools of siRNAs targeting *IFNAR1* and *IFNLR1*, which are expected to neutralize IFN-I and IFN-III signaling, respectively, served as positive controls. Pools of siRNAs targeting *STAT2* were also included in the analysis. Following siRNA transfection, DAOY cells were treated with IFNα2 and Caco2 cells with IFNλ1 for 16 h and then infected with TBEV for another 24 h. Analysis of viral RNA yield by RT-qPCR revealed that, as expected, intracellular viral RNA levels were almost rescued to the level of non-treated cells in the presence of siRNAs targeting IFN receptors and *STAT2* (Fig. 4D). RT-qPCR analyses showed that the four siRNA pools were reducing the expression of their respective target genes, resulting in a 2-fold to 10-fold reduction of mRNA transcripts (Fig. 4E). Reduced expression of *IFI6* significantly enhanced TBEV RNA yields in both cell types as compared to IFN-treated cells transfected with control siRNAs (Fig. 4D), confirming that IFI6 largely contributes to the antiviral state against TBEV, independent of cell or IFN types. Replication of TBEV significantly increased in DAOY cells expressing reduced levels of *KEAP1* and pre-treated with IFNα2 (Fig. 4D). However, it had no apparent effect on viral replication in IFNλ1-treated Caco2 cells (Fig. 4D).

To further investigate the potential effect of *KEAP1* and *IFI6* on TBEV replication, we generated KO cells using lentiviral vectors expressing Cas9 and gRNAs. Caco2 cells expressing non-targeting gRNAs were also produced as control cells. Cells were infected with TBEV and analyzed for the presence of the viral E protein by flow cytometric analysis. As expected from the two screens (Fig. 4B) and siRNA-based validation experiments (Fig. 4D), significantly more IFI6 KO cells were positive for the viral E protein than control cells upon IFNλ1 treatment (Fig. 4F). A significant rescue of viral protein production was also observed in *KEAP1* KO cells (Fig. 4F). However, the increase of infection in *KEAP1* KO cells compared to control cells was independent of prior IFN treatment (Fig. 4G), suggesting that the *KEAP1*-induced antiviral effect is not linked to the IFN-induced antiviral state. Analysis of mRNA levels of *IFI6* and *KEAP1* revealed that *IFI6* was upregulated by IFNα2 and IFNλ1 treatment in both DAOY and Caco2 cells (Fig. 4H), thus qualifying as a genuine ISG in these cell types. However, *KEAP1* mRNA abundance remained unchanged upon treatment with IFNα2 or IFNλ1 in both cell types (Fig. 4H), indicating that it is not an ISG in these two cell types.

The screens thus recovered a set of genes with previously described antiviral functions, including genes crucial for IFN-signaling (*IRF9*, *STAT1,* and *STAT2*), validating our approach. Furthermore, they identified several poorly characterized host factors potentially modulating TBEV infection in human cells (such as TMEM51 and MARCKS) and IFI6 as a potent effector of both IFN families.

### Diminishing *IFI6* expression impairs the antiviral potential of the IFN type I and III responses against TBEV

Other CRISPR/Cas9 screens previously identified IFI6 as a major player in the type I IFN-dependent antiviral defense against several mosquito-borne orthoflaviviruses*,* including ZIKV and YFV (48, 49). So far, its role in IFN-III signaling has not been explored, and its effect on the replication of TBOVs has not been investigated either. Moreover, the mechanism underlying its antiviral activities is poorly understood.

**Fig 5:**
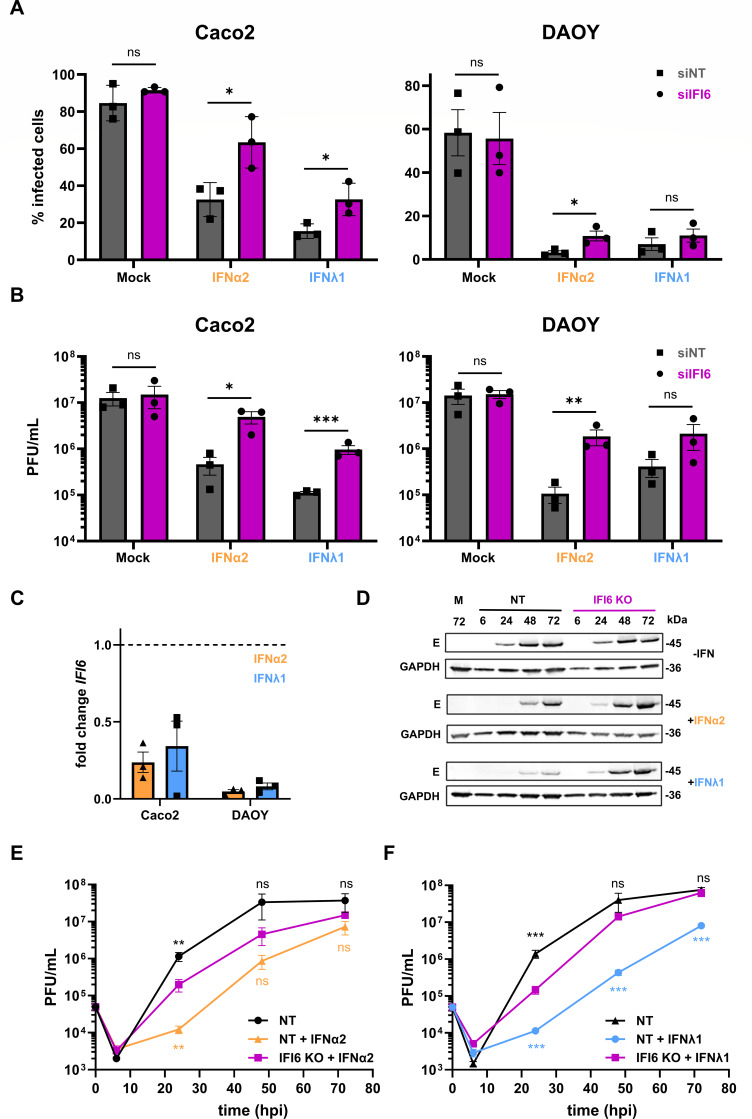
Diminishing IFI6 gene expression impairs the antiviral potential of the IFN type I and III responses against TBEV. (**A**) Cytometry-based analysis of cells stained for orthoflaviviral E protein after siRNA-based knockdown of IFI6 (siIFI6) using a pool of four different siRNAs or non-targeting control (siNT) and subsequent treatment with either IFNα2 (0.25 ng/mL) or IFNλ1 (20 ng/mL) for 16 h before infection with TBEV-Hypr (MOI 1) for 24 h. (**B**) Same experimental setup as panel A, followed by estimating viral titers in the supernatant by plaque assay. (**C**) Reduction of *IFI6* mRNA in Caco2 or DAOY cells transfected with a pool of four siRNAs targeting *IFI6* (siIFI6) expression or non-targeting siRNAs (siNT) and subjected to IFN treatment (IFNα2: 0.25 ng/mL, orange, or IFNλ1: 20 ng/mL, blue) for 16 h, as identified by RT-qPCR analysis. (**D**) Western blot analysis of Caco2 cells with stable knockout of IFI6 (IFI6 KO) or control cells transduced with a lentivirus encoding a non-targeting sgRNA (NT) after treatment with either IFNα2 (0.25 ng/mL) or IFNλ1 (20 ng/mL) for 16 h before infection with TBEV-Hypr (MOI 1) for the indicated times. The abundance of viral protein was determined by staining with an antibody against orthoflaviviral E protein. The data are representative of two independent experiments. (**E**) Plaque assay-based titration of supernatants of Caco2 cells with a targeted knockout of IFI6 (IFI6 KO) or control cells (NT) after treatment with IFNα2 (0.25 ng/mL) for 16 h and infection with TBEV-Hypr (MOI 1) for the indicated time. (**F**) Same experimental setup as panel E but treatment with IFNλ1 (20 ng/mL) for 16 h. Data (**A, B, E, F**) are from three independent experiments ± SEM. ns = non-significant, *P < 0.05, **P < 0.01, ***P < 0.001 by ordinary (**A**), or lognormal (**B**) unpaired, two-tailed *t*-test, or lognormal one-way ANOVA with Dunnett post-test (**E and F**).

To confirm the anti-TBEV function of IFI6 in both IFN-I and -III signaling, a pool of four siRNAs was used to assess the effect of reduced *IFI6* expression on viral replication in Caco2 and DAOY cells after treatment with IFNλ1 or IFNα2. Flow cytometry analysis using anti-E antibody confirmed that IFI6 anti-TBEV activities were dependent on the presence of IFN in both cell lines (Fig. 5A). Similarly, a rescue of the production of infectious virions was observed in cells expressing reduced levels of IFI6, independently of the type of IFN used for pre-treatment and the cell type (Fig. 5B). The siRNA-mediated knockdown reduced the amount of IFI6 mRNA by about 70% in Caco2 cells and by about 90% in DAOY cells, independently of the type of IFN used for pretreatment (Fig. 5C). This validates the used siRNAs as restrictors of *IFI6* expression even in the presence of IFNs.

The effect of IFI6 on viral replication was further assessed in unstimulated and stimulated IFI6-KO Caco2 cells at different time points post-infection. Western blot analysis revealed that the expression of the viral E proteins was increased in cells lacking IFI6, stimulated with IFNλ1 or IFNα2, as compared to control cells that received non-targeting gRNAs (Fig. 5D). In agreement with the western blot analysis, stimulated Caco2 cells lacking IFI6 were producing more infectious viral particles than control cells (Fig. 5E and F). The level was almost rescued to the level of control cells, suggesting that IFI6 is an essential contributor to the innate immune response against TBEV. Together, these data confirm that IFI6 plays a central role in the anti-TBEV signaling induced by both type I and III IFNs.

### IFI6 prevents the replication of diverse tick- and mosquito-borne orthoflaviviruses

**Fig 6:**
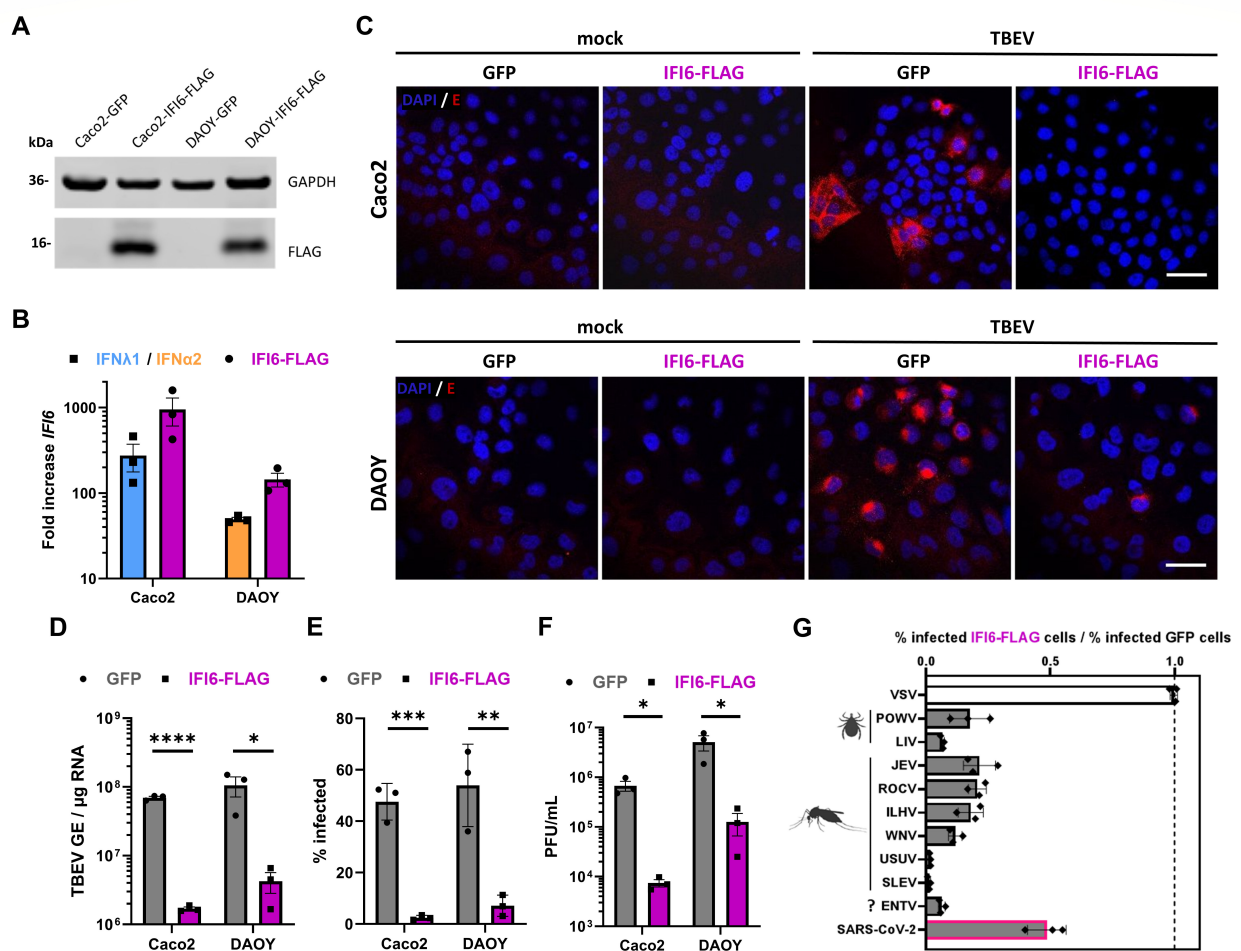
Overexpression of IFI6 prevents replication of diverse tick- and mosquito-borne orthoflaviviruses. (**A**) Western blot analysis of Caco2 or DAOY cells stably expressing IFI6-FLAG or GFP. The abundance of IFI6-FLAG was determined by staining with an antibody against the FLAG protein. The data are representative of three independent experiments. (**B**) Expression levels of *IFI6* mRNA in Caco2 or DAOY cells either stably expressing IFI6-FLAG or after treatment with IFN (Caco2: IFNλ1, 100 ng/mL, blue, or DAOY: IFNα2, 1.25 ng/mL, orange) for 16 h, as identified by RT-qPCR analysis. (**C**) Confocal images of either Caco2 or DAOY cells stably overexpressing GFP or IFI6-FLAG 24 h after mock-infection or infection with TBEV-Hypr (MOI 0.1). Cells were fixed and stained for orthoflaviviral envelope protein (E) to assess their infection status. Images are representative of two independent experiments. Scale bar 30 µm. (**D–F**) Analysis of Caco2 or DAOY cells overexpressing GFP or IFI6-FLAG 24 h after infection with TBEV-Hypr (MOI 0.1) through either RT-qPCR-based estimation of viral genomic copy numbers (**D**), cytometric assessment of cells positive for orthoflaviviral envelope protein (**E**), or plaque assay-based titration to determine the abundance of infectious virions in the supernatant (**F**). (**G**) Caco2 cells overexpressing GFP or IFI6-FLAG were infected with either VSV, orthoflaviviruses, or SARS-CoV-2 (MOIs: VSV:0.01; POWV-SPO:5; LIV:0.1; JEV:1; ROCV:0.1; ILHV:0.1; WNV:1; USUV:1; SLEV:1; ENTV:1; and SARS-CoV-2:0.003) for 24 h, and their percentage of infection was determined through flow cytometry analysis after specific staining for VSV-G, orthoflaviviral envelope, or SARS-CoV-2 nucleocapsid protein. Transmission vectors are indicated for orthoflaviviruses (ticks, mosquitoes, or unknown [?]). Data (**D–G**) are from three independent experiments ± SEM. ns = non-significant, *P < 0.05, **P < 0.01, ***P < 0.001, and ****P < 0.0001 by unpaired, two-tailed *t*-test.

To further characterize the antiviral activity of IFI6, we produced Caco2 and DAOY cell lines stably expressing a C-terminally 3×FLAG-tagged version of IFI6 (IFI6-FLAG), as well as control Caco2 and DAOY cells stably expressing GFP. The expression of IFI6-FLAG in the generated cell lines was detectable by western blot analysis (Fig. 6A). *IFI6* mRNA levels exceeded *IFI6* expression in GFP control cells treated with IFN, although less than 1 log in both cell lines with the concentrations of IFN used (Fig. 6B).

Confocal imaging revealed that expression of IFI6-FLAG resulted in a reduction of cells positive for viral E proteins 24 h post-TBEV infection in both cell lines, as compared to GFP control cells (Fig. 6C). Viral RNA yields were also reduced in DAOY and Caco2 cells expressing IFI6-FLAG (Fig. 6D). In agreement with these results, cytometry analysis showed that around 10 times fewer cells were positive for E proteins in the presence of IFI6-FLAG, as compared to GFP (Fig. 6E). Finally, the release of infectious virions was also significantly reduced in cells expressing IFI6-FLAG (Fig. 6F), further validating the antiviral activity of IFI6.

To evaluate the antiviral effect of IFI6-FLAG on other viruses, we conducted infection experiments with an array of diverse orthoflaviviruses transmitted to humans by ticks (POWV and LIV) or mosquitoes (Japanese encephalitis virus [JEV], Rocio virus [ROCV], Ilheus virus [ILHV], West Nile virus [WNV], Usutu virus [USUV], and St. Louis encephalitis virus [SLEV]). We also included Entebbe bat virus (ENTV), an orthoflavivirus isolated from a bat of the species *Tadarida limbata* whose transmission vector has not been identified yet (50). Moreover, we tested whether IFI6 was active against Vesicular stomatitis virus (VSV), a negative-sense RNA virus belonging to the *Rhabdoviridae* family that replicates in the cytoplasm (51) as a non-orthoflaviviral control. Flow cytometry analysis using an antibody against the viral protein G revealed that VSV replication was not affected by IFI6 expression (Fig. 6G). By contrast, IFI6 significantly reduced the number of E-positive cells in the context of infection with all orthoflaviviruses tested (Fig. 6G). Of note, among the orthoflaviviruses tested, USUV and SLEV were the most sensitive to IFI6 overexpression (Fig. 6G). Finally, we assessed the effect of IFI6 overexpression on SARS-CoV-2 replication since two large-scale loss-of-function screens identified it as a potential SARS-CoV-2 restriction factor (52, 53). The number of cells positive for the viral protein N was reduced by about 50% when IFI6 was overexpressed (Fig. 6G), indicating that IFI6 antiviral activity is not specific to orthoflaviviruses. Our experiments thus indicate that IFI6 can potently restrict the replication of a wide array of orthoflaviviruses, as well as at least one coronavirus, in human cells and acts independently of other ISGs.

### IFI6 is an ER-resident protein that restricts a post-entry step of TBEV replication

**Fig 7:**
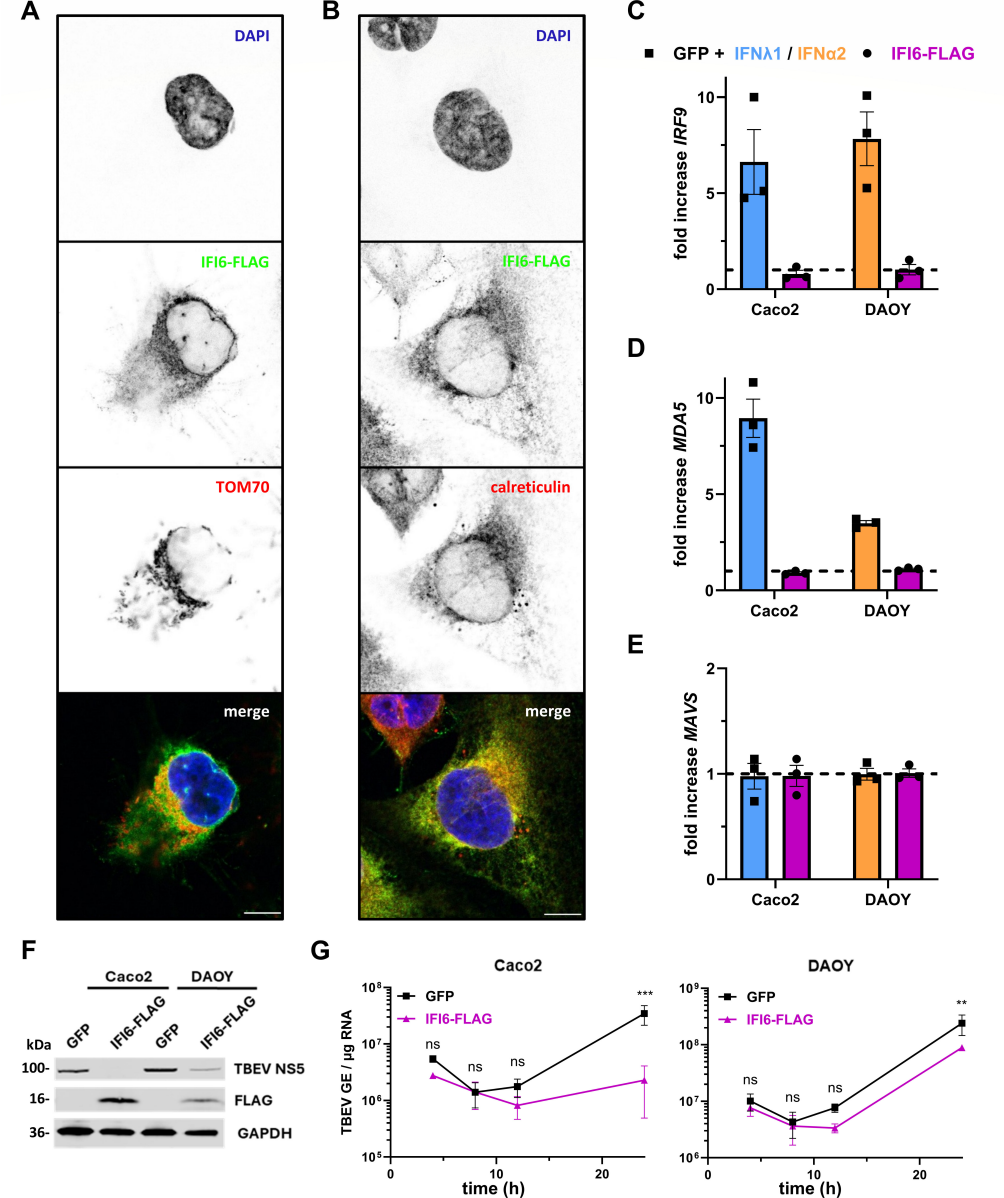
IFI6 is an ER-resident protein that restricts a post-entry step of TBEV replication. (**A and B**) Confocal microscopy analysis of DAOY cells stably overexpressing IFI6-FLAG. Cells were fixed and stained with antibodies for FLAG and mitochondrial marker TOM70 (**A**) or ER-marker calreticulin (**B**). Scale bar, 10 µm. (**C–E**) Analysis of *IRF9* (**C**), *MDA5* (**D**), or *MAVS* (**E**) mRNA levels in Caco2 or DAOY cells ectopically expressing GFP or IFI6-FLAG, via RT-qPCR. GFP-expressing cells were treated with IFN (Caco2: 10 ng/mL IFNλ1, blue; DAOY: 0.1 ng/mL IFNα2, orange) for 16 h. Gene expression was normalized to GAPDH and mRNA levels in mock-treated, GFP-expressing cells. (**F**) Western blot analysis of Caco2 or DAOY cells, either ectopically expressing GFP or IFI6-FLAG, that were electroporated with *in vitro*-synthesized RNA derived from a TBEV Neudoerfl replicon 24 h after electroporation. Whole‐cell lysates were analyzed by staining with antibodies against the indicated proteins. (*N* = 2). (**G**) Caco2 or DAOY cells, either ectopically expressing GFP or IFI6-FLAG, were electroporated with *in vitro*-synthesized RNA derived from a TBEV replicon. At the indicated time points, the relative amounts of viral RNA were measured by RT–qPCR analysis of TBEV-NS5 copy numbers per microgram of total cellular RNA. Data (**C–E and G**) are from three independent experiments ± SEM. ns = non-significant, **P < 0.01, and ***P < 0.001 two-way ANOVA with Sidak correction.

To assess the localization of IFI6 within the cell, we tested several commercial antibodies in different assays. None of them gave us satisfactory results. Previous studies have reported a mitochondrial localization of stably overexpressed IFI6-FLAG in human MCF-7 breast cancer cells (54, 55), while others described IFI6-3×FLAG as an ER-based protein in human hepatoma Huh7.5 cells in which endogenous IFI6 was replaced by a tagged version using CRISPR knock-in approaches (48). Anti-FLAG staining in DAOY cells stably expressing IFI6-FLAG revealed that the antiviral factor colocalized with the ER marker calreticulin, while no overlap in signal was identified with the mitochondrial marker TOM70 (Fig. 7A and B).

A recent report suggested an interaction between swine-IFI6 and the NS4A protein of JEV in human embryonic kidney 293T cells, potentially impeding the involvement of NS4A in remodeling of ER membranes (56). To assess whether TBEV NS4A, or any other viral proteins, could be targeted by IFI6, 293T cells were transfected with FLAG-tagged versions of the 10 individual proteins of TBEV and a C-terminally HA-tagged IFI6. Co-immunoprecipitation assays failed to detect an interaction between viral proteins and IFI6 (Fig. S2), suggesting that IFI6 does not elicit its antiviral potential via an interaction with a viral protein.

Since IFI6 restricts unrelated viruses (Fig. 6G), we assessed whether its activities could be linked to the IFN response. Monitoring the expression levels of three innate immune genes (*IRF9*, *MDA5*, and *MAVS*) illustrated that while *MAVS* was neither influenced by IFN treatment nor IFI6-FLAG expression in Caco2 or DAOY cells, *IRF9* and *MDA5* expression was upregulated by IFNα2 but not influenced by *IFI6* overexpression (Fig. 7C through E). These data suggest that IFI6 does not achieve its antiviral potential by modulating the expression of innate immune genes.

To determine whether IFI6 affects early or late steps of TBEV replication, we took advantage of a replicon generated from the NS protein sequences of the Neudoerfl strain (57). DAOY and Caco2 cells stably expressing either IFI6-FLAG or GFP were electroporated with *in vitro*-synthesized viral RNA generated from the replicon. In this context, viral replication bypasses early steps (viral attachment, entry, fusion in endosomes, and cytoplasmic uncoating). Analysis of the expression of NS5 in cells transfected with the replicon revealed reduced NS5 levels in DAOY cells and an absence of expression in Caco2 cells after 24 h (Fig. 7F). Of note, Caco2 cells expressed more IFI6-FLAG than DAOY cells (Fig. 7F), which could explain the more pronounced effect on NS5 expression. RT-qPCR analysis of viral RNA yields performed at different times post-electroporation revealed that viral replication was reduced after around 24 h in both cell lines expressing IFI6-FLAG compared to GFP-expressing control cells (Fig. 7G). These data indicate that IFI6 acts at a late step of viral replication.

Together, our findings suggest an antiviral effect of IFI6 by suppressing viral genome replication at a post-entry stage, without interacting with viral proteins.

## DISCUSSION

The importance of type I IFN signaling to counteract TBOV replication in human cells is well established (3, 6). In line with our results, the protective effect of pretreatment with type I IFNs was documented for several members of the TBOV family in a variety of cell lines (27, 30, 31, 34). However, to our knowledge, the protective effect of type III IFN pretreatment was only investigated in one study, which states that pretreatment of DAOY cells with IFNλ1 does not protect against TBEV replication (30). This is in stark contrast to our results showing the antiviral potential of IFNλ1 against different TBOVs in two human cell lines. Since the experimental setup did not differ in IFN-type nor concentration, the lack of IFNλ1-induced protection from TBEV in DAOY cells might stem from the 5-fold higher MOI previously used (30), potentially overcoming the IFNλ1-induced antiviral state. Several other studies, however, reported antiviral properties of the IFNλ family against diverse mosquito-borne orthoflaviviruses, suggesting that other members of the genus are also sensitive to the antiviral effects of these cytokines (49, 58–65). The upregulation of type III IFNs following infection with TBOVs was reported in *in vitro* experiments but also in patients infected with TBEV and linked to a milder outcome of disease (30, 39, 66). Furthermore, studies performed on the Polish and Russian populations suggested that, among others, polymorphisms in the *IFNL3/IL28B* gene are associated with a predisposition to severe forms of TBE (39, 67). Treatment of patients with IFNλ is currently successfully explored in the context of a variety of viral infections in humans, at present focusing on hepatic and respiratory viruses (68–74). Compared to type I IFNs, previously used for treatment of viral infections, type III IFNs trigger less expression of inflammatory cytokines, like IL-6 and TNFα (21, 68, 75). IFNλs may thus represent an interesting option to treat patients suffering from neurotropic TBOV infection.

To date, only the impact of type I IFN signaling on TBOV infection has been studied *in vivo* (29, 33, 42, 76, 77). In wild-type mice infected with POWV, death is associated with severe neuroinvasion (78). However, the contribution of type I IFN signaling in regulating POWV infection in mice remains poorly understood. Our data underline the importance of type I IFN signaling in restricting early viral dissemination and delaying lethal POWV-induced disease. The rapid and fulminant disease observed in *Ifnar1^-/-^* and *Ifnar1^-/-^ Ifnlr1^-/-^* mice is unlikely to reflect the typical neurological disease reported in POWV-infected individuals. Instead, it may resemble a systemic sepsis-like syndrome, as previously reported for West Nile virus infection (79). This suggests that early type I IFN responses against POWV promote a fatal trade-off between the control of rapid sepsis-like syndromes and late death by neuroinvasion. The additional depletion of IFN-III signaling did not affect survival in subcutaneously infected C57BL/6J mice, highlighting that, when type I IFN signaling is depleted, the contribution of type III IFN signaling to restricting fulminant POWV disease is undetectable within our experimental framework.

Viral dissemination to the spleen by day 2 has been reported for *Ifnar1^-/-^* C57BL/6J mice intraperitoneally injected with Langat virus (29), consistent with our findings of POWV viral RNA in this tissue at 2 days post-infection. The presence of viral RNA in the liver, although not previously reported in murine models challenged with POWV, aligns with reports of TBOV hepatotropism in patients (80). Notably, subcutaneous infection with POWV resulted in the detection of viral RNA in the small intestine of mice lacking IFN-signaling. This observation is supported by studies showing that TBEV also targets gastrointestinal (GI) tissue in BALB/c or C57BL/6J mice following intravenous or subcutaneous inoculation, respectively, as well as by reports of GI complications in patients during acute TBOV infection (81, 82). The combined depletion of type I and III IFN signaling yielded similar phenotypes to those observed in *Ifnar1^-/-^* C57BL/6J mice, underscoring that type III IFNs do not contribute to limiting POWV replication or viral dissemination when type I IFN signaling is abrogated.

While transmission through ingestion was established for TBEV in a C57BL/6 mouse model and can be observed in patients, it remains unclear whether other TBOVs are able to exploit this way of transmission (33, 83, 84). The only study investigating oral transmission of POWV in animals revealed that infection of a lactating goat with POWV did not result in clinical signs or detectable viremia in the animal but resulted in the development of specific antibodies in its offspring, suggesting milk-borne transmission of POWV (8), which is in line with our data. Moreover, vertical transmission of LGTV through breast milk in A129 mice was reported, and also cases of mother-to-child transmission of TBEV via breast milk were suggested for humans (36). These findings imply that potentially all TBOVs are transmissible from infected mothers to their children during breastfeeding, which needs to be considered for the management of breastfeeding after tick bites in TBOV-endemic areas.

The trend toward earlier lethality in orally infected *Ifnar1^-/-^ Ifnlr1^-/-^* mice, compared to mice lacking only IFN-I signaling, may suggest a contribution of type III IFN-dependent antiviral signaling in the GI tract, as suggested for other viral families (85). However, this trend was statistically non-significant, and no differences in viral titers were detected in the sera of infected *Ifnar1^-/-^* and *Ifnar1^-/-^ Ifnlr1^-/-^* mice at 2 dpi. This aligns with the comparable viral RNA loads observed across diverse tissues in orally infected mice, indicating a negligible role for type III IFN signaling in antiviral defense *in vivo*, regardless of the infection route. Notably, our findings revealed that oral inoculation of POWV results in lower systemic viral titers and reduced viral dissemination compared to footpad inoculation. At 2 days post-infection, only the serum and spleen exhibited robust levels of viral RNA following oral infection, whereas the footpad route led to high viral loads in the spleen, liver, small intestine, and kidneys. Despite this differential dissemination, both inoculation routes resulted in similar lethality in *Ifnar1^-/-^* and *Ifnar1^-/-^ Ifnlr1^-/-^* mice, suggesting that the spleen (and the hematopoietic compartment) may represent a critical disease-defining compartment when type I IFN signaling is abrogated. Wild-type and *Ifnar1^-/-^* C57BL/6J mice inoculated with 10^6^ FFU of TBEV via oral gavage did not exhibit significant mortality, unlike those inoculated via the peroral route at a similar dose (33). These findings may suggest that viral disease and dissemination following oral gavage inoculation are TBO-specific.

The antiviral state induced by IFNs depends on the activation of a plethora of different ISGs, many of which remain uncharacterized (18). While several approaches successfully identified IFN-I effectors against mosquito-borne orthoflaviviruses (48, 49, 86–88), effectors against TBOVs are poorly described. Our parallel CRISPR/Cas9-KO screening approach recovered factors previously identified as antiviral effectors against TBOVs, such as *IFITM1* and *IFITM3* (41). Moreover, with *IARS*, *MED12*, *CHORDC1,* and *COMMD3* for the type I screen and *TMEM51*, *CDH1*, *JUN*, *SLC16A1*, *RASAL2,* and *HES1* for the type III screen, we identified several novel genes with potential involvement in the IFN-induced cellular defense mechanism against TBOVs. Further investigations would be required to validate the antiviral activities of these candidates and decipher their mechanisms of action. Of note, we did not identify *RSAD2* (viperin) or *TRIM5α,* two ISGs previously reported to counteract TBOV infection in human A549 and/or HEK293T cells, although they were included in the sgRNA library we screened (44, 89–92). Their antiviral function may be cell type-specific. The screens further suggested a proviral effect of *USP18*, *ISG15,* and *VMP1*, which agrees with previous experiments performed in human cells infected with other members of the *Flaviviridae* family (46, 47). *KEAP1* emerged as a potential IFN-I and IFN-III effector against TBEV. Although further investigations revealed that its action is IFN-independent in Caco2 and DAOY cells, it may be induced by IFNs in other cell types.

Our screens identified *IFI6* as an IFN-I and IFN-III effector for the anti-TBEV response in human cells. Reducing IFI6 expression in the presence of IFN-I/-III enhanced TBEV replication to a level comparable to the inhibition of IFNAR1 or STAT2, suggesting that it largely contributes to the innate immune response against TBEV. We showed that IFI6 restricts the replication of orthoflaviviruses transmitted by ticks, *Culex,* and *Aedes* mosquitoes. In line with previous reports (48), our data support the hypothesis of IFI6 being an ER resident and suppressing the replication of orthoflaviviruses at a post-entry step. We observed an anti-SARS-CoV-2 effect of IFI6 expression in Caco2 cells, which was unexpected since the replication of OC43, a related betacoronavirus, was unaffected by IFI6 overexpression in Huh7.5 cells (49). These effects on SARS-CoV-2 replication thus challenge the idea of IFI6 solely preventing the formation of orthoflavivirus-specific alterations in the ER membrane (48). IFI6 may thus restrict the replication of unrelated viruses that replicate in the ER, preventing the formation of virally induced ER remodeling (48), a process that is key for orthoflaviviral and betacoronaviral replication (93–95). This re-arrangement of ER membrane structures is mostly driven by several viral proteins, although the detailed mechanisms of their involvement are yet to be defined (96). How the restructuring of the ER membranes by viral proteins might be influenced by IFI6, however, remains unclear. A direct interaction between the 2K protein of JEV, which lies in the last transmembrane domain of NS4A, and swine IFI6 (sIFI6) was recently suggested (56). Cleavage of the 2K protein from NS4A through the viral NS2B-NS3 protease modulates its potential to induce membrane rearrangements (97, 98). This is further illustrated by a mutation in the genome of WNV that prevents cleavage of NS4A-2K, completely abrogating viral replication (99). IFI6 may thus block the 2K cleavage and prevent the ER-remodeling potential of the NS4A protein of orthoflaviviruses. However, we did not detect interaction between human IFI6 and TBEV NS4A-2K, or any other viral protein. Furthermore, IFI6 was not able to prevent NS4A-2K cleavage or any other polyprotein processing events induced by the viral protease (48, 49). Together, these results challenge the idea of IFI6 exercising its antiviral potential by inhibiting viral protease activity and subsequent prevention of ER remodeling. This makes further investigation of the mechanisms underlying IFI6 antiviral activities indispensable. Importantly, this is hindered by the fact that no murine ortholog of IFI6 exists, greatly complicating its investigation in an *in vivo* context.

## MATERIALS AND METHODS

### Mice

All mice were maintained in facilities accredited by the Association for the Assessment and Accreditation of Laboratory Animal Care (AAALAC). All replication-competent Powassan Virus experiments were performed in a biosafety level 3 laboratory (BSL-3) at the Boston University National Emerging Infectious Diseases Laboratories (NEIDL).

C57BL/6J (Cat. #000664) and B6(Cg)-Ifnar1tm1.2Ees/J (*Ifnar1^-/-^* mice; Cat. #028288) mice that are deficient for type I innate immune signaling were acquired from Jackson Laboratories. *Ifnar1^-/-^ Ifnlr1^-/-^* mice that are deficient in type I and III innate immune signaling were kindly provided by Sergei Kotenko at Rutgers University. All mice were maintained at the Animal Science Center at Boston University prior to being moved to the NEIDL for experiments.

Mice in the NEIDL BSL-3 facility were group-housed by sex in Tecniplast green line individually ventilated cages (Tecniplast). Mice were maintained on a 12:12 light cycle at 30%–70% humidity and provided standard water and standard chow diets (LabDiet).

### Cell lines and viruses

Human colorectal adenocarcinoma Caco2-TC7 cells (American Type Culture Collection [ATCC], HTB-37), human medulloblastoma DAOY cells (ATCC, HTB-186), human embryonic kidney (HEK) 293T cells (ATCC, CRL‐3216), human glioblastoma U87-MG cells (ATCC, HTB-14), African green monkey kidney epithelial Vero cells (ATCC), baby hamster kidney BHK-21 cells (ATCC), and human hepatoma-derived Huh7.5 cells (ATCC) were maintained in Dulbecco’s-modified Eagle’s medium (DMEM) (Gibco) containing GlutaMAX I and sodium pyruvate (Invitrogen) supplemented with 10% heat‐inactivated fetal bovine serum (FBS) (Dutscher) and 1% penicillin and streptomycin (10,000 IU/mL; Thermo Fisher Scientific) at 37°C and 5% CO2.

Experiments with TBEV, POWV, LIV, ZIKV, JEV, ROCV, ILHV, WNV, USUV, SLEV, ENTV, and SARS-CoV-2 were performed in a BSL‐3 laboratory, following safety and security protocols approved by the risk prevention service of the Institut Pasteur, Paris, or the NEIDL at Boston University. Experiments with VSV and lentiviruses were performed in a BSL-2+ setting following biosafety regulations of the Institut Pasteur, Paris. VSV Indiana was kindly provided by N. Escriou (Institut Pasteur, Paris). The TBEV-Hypr (001v-EVA134), POWV-LB (001v-EVA124), ROCV (001v-EVA126), ILHV (001v-EVA106), and ENTV (001v-EVA84) were obtained from the European Virus Archive (EVAg; https://www.european-virus-archive.com/). POWV SPO (TVP20467), also known as Deer Tick Virus, was a kind gift from Robert B. Tesh (UTMB, Arbovirus Reference Collection, World Reference, Center of Emerging Viruses and Arboviruses, Galveston, TX). Louping Ill virus (strain LI 3/1; APHA reference Arb126) was a kind gift from Nick Johnson (Animal and Plant Health Agency, Addlestone, Surrey, UK). JEV (strain RP9), SLEV (strain MSI-7), and WNV (strain IS-98-STI) were provided by the biological resource center of the Institut Pasteur. USUV (strain D18-03348) was a kind gift from Nolwenn Dheilly (Institut Pasteur, Paris, France). SARS-CoV-2 (strain BetaCoV/France/IDF0372/2020) was supplied by the French National Reference Center for Respiratory Viruses hosted by Institut Pasteur (Paris, France). Vero cells were used to produce all viral stocks, with the exception of POWV SPO stocks, which were produced on BHK-21 cells. Vero cells were used to assess the number of infectious virions produced through plaque assay, as previously described (100). Only the POWV-LB virus used for infection of mice was titrated using a focus-forming assay on Vero cells. Briefly, Vero cell monolayers in a 24-well plate were infected with serial dilutions of virus stock for 2 h at 37°C; then, the supernatant was replaced with a 1:1 mixture of 2× DMEM and CMC and incubated for 6 days at 37°C under a 5% CO2 atmosphere. The overlay was then removed; the cells were washed with PBS, fixed with 4% PFA at RT for 15 min, and then permeabilized and blocked with PBS (1%BSA, 1% Triton-X) at RT for 1 h. Cells were then incubated with PBS (1% BSA) with a rabbit pan-orthoflaviviral E protein antibody (1:2,000, Thermo Fisher, NBP52666549) at RT for 1 h while shaking. Monolayers were then washed 3× with PBS and incubated with PBS (1% BSA) holding Li-Cor IRDye 800CW anti-rabbit antibody (1:5,000, Fisher, NC9523609) for 45 min. Cells were washed 3× with PBS and plates imaged using an Odyssey DLx machine (Li-Cor). Viral titers were calculated according to the dilution and plated volume. All indicated MOIs thus refer to Vero cell MOIs.

### Antibodies and cytokines

The following primary antibodies were used: commercially available antibodies against β‐actin (produced in mouse, clone AC‐74), FLAG (mouse, M2 F3165‐1MG), 4G2 pan‐orthoflavivirus (mouse, 1:1,000, kind gift from Philippe Desprès), anti-TBEV NS5 (34), TOM70 (rabbit, ab289977), calreticulin (rabbit, AB2907), and HA (rabbit, H6908) were obtained and used for immunoblotting at the indicated dilutions. Furthermore, FLAG M2 was used at 1:2,000, 4G2 at 1:2,000, and anti-NS5 at 1:1,000 dilutions for immunofluorescence staining for microscopy analysis. 4G2 pan‐orthoflavivirus antibody was used at 1:1,000, anti-VSV-G antibody (kind gift from Gert Zimmer) was used at 1:2,000, and anti-SARS-CoV-2-NCP-1 antibody (kind gift from Olivier Schwartz [101]) was used at 1:200 dilutions in flow cytometry, respectively.

For secondary staining, the following commercially available antibodies were employed: Alexa Fluor 488 goat anti‐mouse IgG (H+L, A11001), Alexa Fluor 647 donkey anti‐mouse IgG (H+L, A31571), Alexa Fluor 647 goat anti‐rabbit IgG (H+L, A21244), Alexa Fluor 680 goat anti‐mouse IgG (H+L, A21058), goat anti‐chicken IgY (H+L) Dylight 800 (SA5‐10076), and goat anti‐rabbit IgG (H+L) Dylight 800 (SA5‐35571). All secondary antibodies were used at a 1:1,000 dilution for cytometry and immunofluorescence assays and diluted at 1:10,000 for western blot analysis.

Human recombinant IFNα2 (PBL Biosciences) and IFNλ1 (IL-29, Invitrogen) were used to stimulate cells at the concentrations (diluted in DMEM 10% FBS; 1% P/S) and for the time indicated in the respective figure legends.

### Viral infections

Cells were infected at MOIs determined on Vero cells (as indicated in the respective figure legends) by a 2 h incubation in DMEM medium containing 2% FBS at 37°C under a 5% CO2 atmosphere. Cells were then washed with PBS (Gibco), the infection medium was replaced by DMEM (10% FBS; 1% P/S), and the cells were subsequently incubated at 37°C under a 5% CO2 atmosphere until collected for analysis at the indicated timepoint.

### Transfections

293T cells were transfected using the *Trans* IT‐293 (Mirus) reagent following the protocol provided by the manufacturer. For co‐immunoprecipitation experiments, 5 × 10^5^ cells in 12‐well plates were transfected with 250 ng of each plasmid, as indicated in the figure.

The ON-TARGETplus siRNAs used for either screen validation or IFI6 knockdown experiments in DAYO and Caco2 cells (Table S1) were purchased from Horizon. Caco2 and DAOY cells were seeded on a 12-well plate (8 × 10^4^/well) and transfected with Lipofectamine RNAiMAX transfection reagent (Thermo Fisher Scientific) according to the manufacturer’s instructions.

### Immunoblot and immunoprecipitation analyses

Cells were lysed using radioimmunoprecipitation assay (RIPA) buffer (Sigma‐Aldrich), supplemented with a protease and phosphatase inhibitor cocktail (Roche). Subsequently, samples were denatured in 4× protein sample loading buffer (Li‐Cor Bioscience) under reducing conditions (NuPAGE reducing agent, Thermo Fisher Scientific) when non-infected or not when infected with orthoflaviviruses. Only 293T cells expressing TBEV NS2A were lysed using the Mem‐PER plus membrane protein extraction kit (Thermo Fisher Scientific) and sonicated for 20 min at 100% amplitude and 2/2 pulse. Proteins were then separated by SDS-PAGE (NuPAGE 4%–12% Bis‐Tris gel, Invitrogen) and transferred to nitrocellulose membranes (Bio‐Rad) utilizing a *Trans*‐Blot Turbo Transfer system (Bio‐Rad). Membranes were blocked with PBS‐0.1% Tween 20 (PBS‐T) containing 5% milk. Following blocking, membranes were incubated for 1 h at RT or overnight at 4°C with primary antibodies diluted in blocking buffer. Subsequently, membranes were washed 3× with PBS-T and incubated for 45 min at room temperature with secondary antibodies (anti‐rabbit/mouse IgG (H+L) DyLight 800/680) diluted in blocking buffer, followed by 3× washing of the membranes with PBS-T. Images were acquired using an Odyssey CLx infrared imaging system (Li‐Cor Bioscience).

For co‐immunoprecipitation analysis, a portion of the cell lysates was incubated overnight with magnetic beads coupled with anti‐FLAG M2 (Sigma‐Aldrich, M8823). Following incubation, immunoprecipitates were washed four times with washing buffer and analyzed by immunoblot as described above.

### RNA extraction and RT-qPCR analyses

Total RNA was extracted from cell lysates utilizing the NucleoSpin RNA II kit (Macherey‐Nagel) or the NucleoMag RNA kit (Macherey-Nagel) according to the manufacturer’s instructions and subsequently eluted in nuclease‐free water. First‐strand cDNA synthesis involved 1 μg of total RNA using the RevertAid H Minus Moloney murine leukemia virus (M‐MuLV) reverse transcriptase (Thermo Fisher Scientific) with random primers p(dN)6 (Roche). Quantitative real‐time PCR was conducted on a Quant Studio 6 Flex real-time PCR system (Applied Biosystems) using SYBR green PCR master mix (Life Technologies). Data were analyzed using the ΔΔCT method, with glyceraldehyde‐3‐phosphate dehydrogenase (GAPDH) serving as the normalization reference for human samples. Technical triplicates were performed for each experiment. RT-qPCR primer sequences are detailed in Table S2. Quantification of TBEV and POWV genomes was achieved by extrapolation from a standard curve derived from serial dilutions of plasmids encoding TBEV NS5 (pCi‐Neo‐NS5 TBEV) or POWV NS5 (pCi-Neo-NS5 POWV).

### Immunofluorescence assays

Cells were fixed with 4% paraformaldehyde (PFA) (Sigma‐Aldrich) at RT for 30 min, followed by permeabilization with PBS containing 0.5% Triton-X at RT for 15 min. Subsequently, cells were blocked for 30 min with PBS containing 0.05% Tween and 5% BSA, before being exposed to the specified primary antibodies diluted in PBS containing 0.05% Tween and 2% BSA for 1 h. Following antibody incubation, cells were washed 3× with PBS containing 0.05% Tween. Secondary Alexa Fluor 488- or 647-conjugated antibodies diluted in PBS containing 0.05% Tween and 2% BSA were then applied for 1 h. After secondary antibody incubation, cells were washed 3× with PBS containing 0.05% and once with PBS alone. Nuclei were stained for 15 min using PBS/NucBlue (Life Technologies). Following staining, slides were mounted with Prolong Gold imaging medium (Life Technologies). Images were captured utilizing a Leica SP8 confocal microscope.

### Flow cytometry

Infected cells underwent fixation using the Cytofix/Cytoperm fixation and permeabilization kit (BD Pharmingen) for 20 min, followed by three washes with the corresponding wash buffer. Subsequently, cells were stained with the anti‐E mAb 4G2 primary antibody, diluted in wash buffer, at 4°C for 1 h. After another three washes with wash buffer, cells were stained with secondary Alexa 488 or Alexa 647 antibody for 45 min in the dark at 4°C. Data acquisition was performed using the Attune NxT Acoustic Focusing Cytometer (Life Technologies), and analysis was conducted using FlowJo v10.8.1 software.

### Generation of ISG-KO library cells

In total, 26-well plates were seeded with 4 × 10^6^ HEK293T cells in DMEM (10% FBS, 1% P/S). After 24 h, cells were simultaneously transfected with the 667 ng lentiCRISPRv2 ISG library plasmids (https://www.addgene.org/pooled-library/lenticrisprv2-isg-library/), 500 ng plasmids coding for HIV Gag/Pol (psPAX2), and 333 ng of plasmids encoding for the VSVg envelope (pVSV-G opt) per well using *Trans* IT‐293 (Mirus) transfection reagent following the manufacturer’s instructions. Transfection medium was replaced after 24 h, and supernatants were harvested 24 and 48 h later, filtered, concentrated by ultracentrifugation (22,000 × *g*, 4°C for 1 h), and pooled. To generate the ISG KO library cells, 3 × 10^7^ Caco2 or DAOY cells were seeded in 12-well plates 24 h before transduction. Each well was then transduced at an MOI of 1 as identified by colony formation titering assays for lentiviruses on Caco2 or DAOY cells following previously described protocols (102). Briefly, concentrated lentivector was diluted in serum-free DMEM, supplemented with 20 µg/mL of DEAE dextran (Sigma, D9885), and cells were spin-infected for 30 min at 1,100 × *g*. After 48 h, transduced cells were selected by puromycin treatment for 10 days (Caco2: 10 µg/mL, DAOY: 4 µg/mL; Sigma, P8833).

### CRISPR/Cas9 screens

In total, 1.3 × 10^7^ Caco2 or DAOY cells were treated with IFNλ1 (Caco2, 100 ng/mL) or IFNα2 (DAOY, 0.5 ng/mL). After 16 h, cells were infected with TBEV-Hypr at an MOI of 1 in DMEM (2% FBS, 1% P/S). After 2 h, the viral inoculum was removed, washed once with PBS, and cells were maintained in DMEM containing 10% FBS and 1% P/S for 24 h. Thereafter, cells were collected and fixed using the fixation buffer supplied with the Cytofix/Cytoperm fixation and permeabilization kit (BD Pharmigen). Fixed cells were washed in cold Cytoperm buffer and then incubated for 1 h at 4°C under rotation with primary antibody (4G2, mouse, 1:1,000) in the same buffer. Incubation with the secondary antibody (Alexa 488 anti-mouse, 1:10,000) was performed for 45 min at 4°C under rotation. Stained cells were resuspended in cold sorting buffer containing PBS, 2% FBS, 25 mM Hepes, and 5 mM EDTA. Cells were sorted into infected and non-infected cells using a BD FACS Aria Fusion machine. Sorted and control (non-infected, not IFN-treated) cells were then centrifuged for 20 min at 2,000 × *g* and resuspended in lysis buffer (NaCl 300 mM, SDS 0.1%, EDTA 10 mM, EGTA 20 mM, Tris 10 mM) supplemented with 1% Proteinase K (Qiagen) and 1% RNAse A/T1 (Sigma) and incubated overnight at 65°C. DNA was isolated with two consecutive phenol-chloroform extractions and recovered by ethanol precipitation. Next, a PCR reaction using barcoded primers to allow for demultiplexing of the samples after sequencing was performed using the Herculase II Fusion DNA Polymerase (Agilent) and DNA oligos as described in a previous study (2). The resultant PCR products were purified using a QIAquick PCR Purification kit (Qiagen, 28104) and subjected to a second PCR following primer designs described in (102) before being purified using Agencourt AMPure XP Beads (Beckman Coulter Life Sciences). Next-generation sequencing was performed utilizing the NextSeq 500/550 High Output Kit v2.5 with 75 cycles (Illumina).

### Screen analysis

Reads were demultiplexed using quality-aware fastq demultiplexer v0.1.1. Sequencing adapters were removed using Trimmomatic v0.39. The reference library was built using Bowtie2 v2.3.5.1. Read mapping was performed with bowtie2 and samtools v2.0.4. Mapping analysis and gene selection were performed using MAGeCK v0.5.9, normalizing the data with default parameters.

### Generation of cells with stable KO of IFI6 or KEAP1

Stable KO of IFI6 or KEAP1 in Caco2 or DAOY cells was achieved through transduction with lentivectors harboring sgRNA sequences targeting the specific genes (Table S3) introduced into the lentiCRISPRv2 backbone. The sgRNAs were produced by hybridization of oligonucleotides encoding for the respective sgRNA sequence flanked by sequences resembling restriction sites for the BsmBI enzyme. LentiCRISPRv2 plasmids and the hybridized oligonucleotides were digested with the restriction enzyme and gel purified. The components obtained were then ligated using a T4 ligase for 2 h at RT. The resultant plasmids were transformed into Stbl3 bacteria for amplification and sequenced to ensure the successful incorporation of the sgRNA sequence. After, HEK293T cells plated in a 6-well plate and grown to 70% confluency were simultaneously transfected with the 667 ng PIKA sgRNA library plasmids, 500 ng plasmids coding for HIV Gag/Pol (psPAX2), and 333 ng of plasmids encoding for the VSVg envelope (pVSV-G opt) per well using *Trans* IT‐293 (Mirus) transfection reagent following the manufacturer’s instructions. Transfection medium was replaced after 24 h, and supernatants were harvested 24 and 48 h later, filtered, and pooled. To generate the KO cells, a 6-well plate with Caco2 or DAOY cells was incubated with lentivector-holding supernatant diluted 1:2 in serum-free DMEM for 2 h before replacing the inoculum with DMEM (10% FBS, 1% P/S). After 48 h, transduced cells were selected by puromycin treatment for 10 days (Caco2: 8 µg/mL, DAOY: 4 µg/mL; Sigma, P8833).

### Generation of IFI6-Flag expression plasmid and lentiviral vectors

IFI6-Flag expression plasmid was generated by cloning the IFI6 coding sequence from IFN-stimulated DAOY cells’ cDNA into p3XFLAG-CMV−14 vector (Sigma) using EcoRI/KpnI restriction sites (Fwd: 5′-TCTAGTGAATTCCCACCATGCGGCAGA AGGCGGTATCGC −3′, Rev: 5′-GACTGGTACCTCCTCATCCTCCTCACTATC-3′).

The generated IFI6-3× FLAG plasmid was used to transfer the IFI6-3× FLAG cassette into the pFlap-Ubc-nLuc-IRES-Puro lentiviral vector (kind gift of Pierre Charneau). PCR primers were designed to add BclI/XhoI restriction sites to IFI6-3× FLAG coding sequence (Fwd: 5′-CTCTAGAGGATCCCGGGCTG-3′, Rev: 5′-GAGAGGCTCGAGTCTCACTACTTGTCATCGTCA-3′), and the amplicon was cloned into pFlap-Ubc-IRES-Puro lentiviral vector digested with BamHI/XhoI to generate the pFlap-Ubc-IFI6-3× FLAG-IRES-Puro plasmid. Amplicon was cloned in pFlap-Ubc-IRES-Puro using BamHI/XhoI restriction sites.

The HA-tagged version of IFI6 was obtained by amplifying IFI6 from the IFI6-3× FLAG plasmid, with primers designed to add BclI/XhoI restriction sites along with a C-terminal HA tag (Fwd: 5′- TCTAGTTGATCAGCCACCATGCGGCAGAAGGCGGT-3′, Rev: 5′-GAGAG GCTCGAGTCACTAAGCGTAATCTGGAACATCGTATGGGTAAGCCCGGGATCCTCTAGAGTC-3′), and amplicon was cloned into pFlap-Ubc-IRES-Puro lentiviral vector digested with BamHI/XhoI to generate the pFlap-Ubc-IFI6-HA-IRES-Puro plasmid used for transfection.

Control GFP-expressing lentiviral vector was obtained using the same cloning strategy, amplifying GFP from pEGFP-C1 plasmid (Clontech) (Fwd: 5′-TCTAGTTGATCAGCCACCATGGTGAGCAAGGGCGA-3′, Rev: 5′-GAGAGGCTCGAGTCTTACTTGTACAGCTCGTCCAT-3′). All plasmids were propagated in Stbl3 *E. coli* cells and verified by sequencing (Eurofins Genomics).

### Generation of stable cell lines

HEK293T cells plated in a 6-well plate and grown to 70% confluency were simultaneously transfected with 1.6 µg of either p6.16-Lenti-GW-TMEM51, pFlap-Ubc-IFI6-3xFLAG-IRES-Puro pFlap-Ubc-IFI6-HA-IRES-Puro, or pFlap-Ubc-EGFP-IRES-Puro, 1 µg plasmids coding for HIV Gag/Pol (psPAX2) and 400 ng of plasmids encoding for the VSVg envelope (pVSV-G opt) per well using *Trans* IT‐293 (Mirus) transfection reagent following the manufacturer’s instructions. Transfection medium was replaced after 24 h, and supernatants were harvested 24 and 48 h later, filtered, and pooled. To generate the cells stably expressing IFI6-FLAG, a 6-well plate with Caco2 or DAOY cells was incubated with lentivector-containing supernatant diluted 1:2 in serum-free DMEM for 2 h before replacing the inoculum with DMEM (10% FBS, 1% P/S). After 48 h, transduced cells were selected by zeocin treatment for TMEM51-expressing cells (Caco2: 300 µg/mL, DAOY: 200 µg/mL, Sigma) or puromycin treatment for IFI6-3× FLAG or IFI6-HA and GFP expressing cells (Caco2: 10 µg/mL, DAOY: 4 µg/mL, Sigma) for 10 days.

HEK293T cells plated in a 6-well plate and grown to 70% confluency were simultaneously transfected with 1.6 µg of either p6.16-Lenti-GW-TMEM51, pFlap-Ubc-IFI6-3× FLAG-IRES-Puro, or pFlap-Ubc-EGFP-IRES-Puro, 1 µg plasmids coding for HIV Gag/Pol (psPAX2). and 400 ng of plasmids encoding for the VSVg envelope (pVSV-G opt) per well using *Trans* IT‐293 (Mirus) transfection reagent following the manufacturer’s instructions. Transfection medium was replaced after 24 h, and supernatants were harvested 24 and 48 h later, filtered, and pooled. To generate the KO cells, a 6-well plate with Caco2 or DAOY cells was incubated with lentivector-containing supernatant diluted 1:2 in serum-free DMEM for 2 h before replacing the inoculum with DMEM (10% FBS, 1% P/S). After 48 h, transduced cells were selected by zeocin treatment for TMEM51-expressing cells (Caco2: 300 µg/mL, DAOY: 200 µg/mL, Sigma) or puromycin treatment for IFI6-3× FLAG and GFP expressing cells (Caco2: 10 µg/mL, DAOY: 4 µg/mL, Sigma) for 10 days.

### *In vitro* transcription and electroporation of TBEV replicons

TBEV pTND/∆ME replicon plasmids (kindly provided by Franz X. Heinz via Karin Stiasny) (57) were linearized via NheI digestion and blunt-ended using the Quick Blunting Kit (New England Biolabs); 5 µg of purified DNA template were utilized for T7-mediated *in vitro* transcription employing the RiboMAX large‐scale RNA production system T7 (Promega), with the addition of 40 mM cap analog (Ribo m7G Cap, Promega) as proposed by the manufacturer’s protocol. After treatment with RQ1 DNase (Promega), RNA was purified using an RNA clean-up kit (Macherey‐Nagel). *In vitro* synthesized TBEV replicon RNA was then delivered into Caco2 or DAOY cells via electroporation. Here, 2 × 10^6^ cells were trypsinized, washed 3× in cold PBS, resuspended in 200 µL cold PBS, and electroporated with 5 μg of RNA in 0.4-cm electroporation cuvettes (Biorad), using a 950 μF and 260 V pulse with the Genepulser system (Biorad). Following electroporation, cells were collected in 3.2 mL of warm medium, split into four wells of a 12-well plate, and incubated at 37°C under standard conditions.

### Mouse infection and monitoring

C57BL/6J, *Ifnar1^-/-^*, and *Ifnar1^-/-^ Ifnlr1^-/-^* male and female adult mice (9–20 weeks of age) were infected through subcutaneous injection in the foodpad with POWV-LB (10^3^ FFU) diluted in 50 µL of PBS or through oral gavage with POWV-LB (10^5^ FFU). Clinical manifestations of disease were monitored daily, and signs of clinical disease progression were recorded through weighing, clinical scoring, and temperature measurements using a UID temperature implantable probe (Unified Information Devices; SKU: UCT-2112-198). Overall appearance was assessed using a clinical scoring matrix assigned after assessing the following parameters: significant (10%–24%) body weight loss, neurological signs (hind limb paralysis, weak grip, and ataxia), appearance (ruffled fur or hunched), responsiveness (low to moderate), and behavior (circling or head tilt). Mice that scored higher than three on 2 consecutive days were euthanized and documented as dead for the following day.

### Tissue collection

At 2 days postinfection, mice were anesthetized using 1%–3% isoflurane, followed by euthanasia using an overdose of ketamine/xylazine. Whole blood (200 µL) was collected from the heart of mice and transferred into Eppendorf tubes. Serum was collected from blood by centrifugation at 3,500 RPM and 4°C for 10 min and stored at −80°C for later analysis; 20–30 mg of liver, small intestine, brain, spleen, and kidney tissue were weighed and transferred into Eppendorf tubes holding 600 µL RNAlater (Ambion) and stored at 4°C for next day processing or at −80°C for future processing.

### RNA extraction from serum and tissues

The isolated tissues (20–30 mg) were transferred into 2 mL tubes containing buffer RLT–1% β-mercaptoethanol (Qiagen) and a 5-mm stainless steel bead. Tissues were lysed using a TissueLyser (Qiagen) at 30 cycles/s for 2 min, 1 min wait, 30 cycles/s for 2 min, and centrifuged at high speed (13,000 rpm) for 10 min. Total RNA was then extracted from the resulting supernatant or serum using a RNeasy minikit (Qiagen) following the manufacturer’s instructions.

### Serum titrations

Viral particle quantification in sera was performed by plaque assay on HuH7.5 cells; 4.5 × 10^4^ cells were plated in 48-well plates 16–24 hours prior to experiments. For titration, sera were diluted 1:10 in OptiMEM + GlutaMax (Gibco) and then serially diluted (10^−1^ – 10^−8^); 100 μL of each dilution was plated onto HuH7.5 cells, incubated for 2 h at 37°C and 5% CO_2_, and then, the inoculum was removed prior to overlay of 800 μL of 1% Methylcellulose media in DMEM 10% FBS. Plates were incubated for 5 days at 37°C and 5% CO_2_ before removal of overlay and fixation with 10% neutral-buffered formalin. After 2 h of fixation, formalin was removed, and the fixed cells were stained with 0.1% crystal violet in 10% ethanol for 30 min. Cells were then washed, and plaques were counted.

### Enzyme-linked immunosorbent assays (ELISA)

Mice sera were analyzed by ELISA to determine the amounts of secreted IFNα with the ELISA MAX Deluxe Set Mouse IFN-α1 (BioLegend, Cat. #447904). Sera samples were diluted 1:4 in 1× Assay Diluent, and 50 μL of diluted sera was processed according to the manufacturer’s protocol. ELISA assays were run using Biolegend Nunc MaxiSorp ELISA Plates (Biolegend Cat #423501) and analyzed with a Varioskan LUX Multimode Microplate Reader (Thermofisher Scientific, Waltham, MA, USA).

### Data representation and statistical analyses

Data are presented and analyzed using GraphPad Prism software v10.0.5. Significance was calculated as indicated in the corresponding figure legend. Where not indicated, no statistical significance was determined.

## Data Availability

All data necessary to evaluate the conclusions in this study are provided in the main text and supplemental material. The sequencing data generated during the study have been deposited in the NCBI Gene Expression Omnibus (GEO) under accession number GSE310333.

## SUPPLEMENTAL MATERIAL

Fig. S1 jvi.00760-25-s0001.tif ELISA *in vivo*.

Fig. S2 jvi.00760-25-s0002.tif co-IP viral proteins/IFI6.

Supplemental material jvi.00760-25-s0003.docx Legends for Fig. S1 and S2; Tables S1 to S3.

ASM does not own the copyrights to Supplemental Material that may be linked to, or accessed through, an article. The authors have granted ASM a non-exclusive, world-wide license to publish the Supplemental Material files. Please contact the corresponding author directly for reuse.
