# Supplementary figures and images for: Type I and III interferon responses restrict infection by tick-borne orthoflaviviruses through IFI6
Source: J Virol. 2026 Apr 14;100(5):e00760-25. doi: 10.1128/jvi.00760-25 (PMC13185562; doi:10.1128/jvi.00760-25)

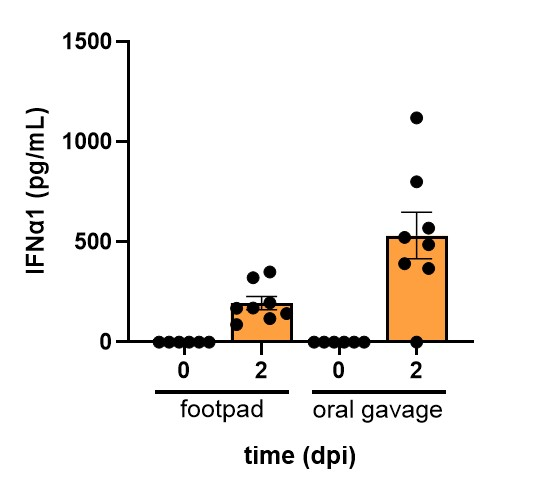

Supplement: Fig. S1 — ELISA in vivo. [file jvi.00760-25-s0001.tif]

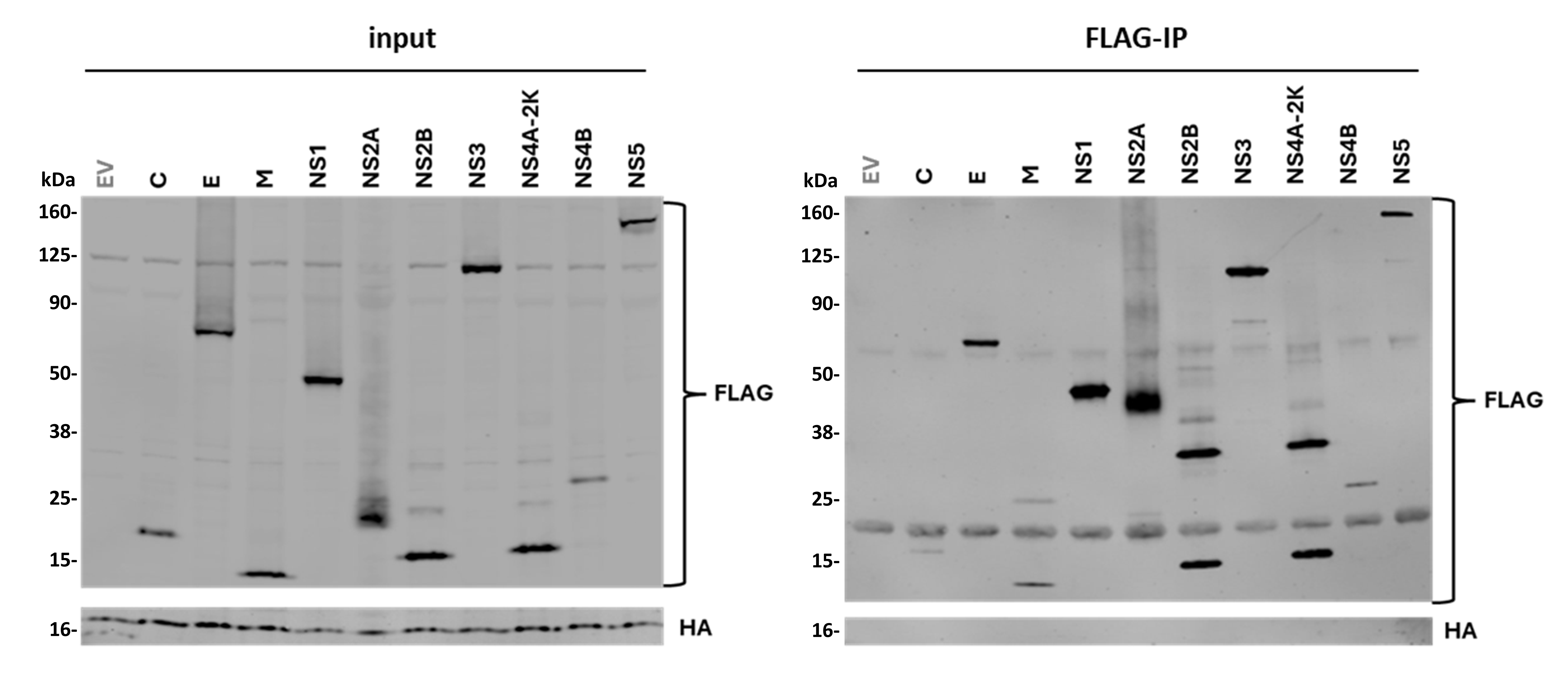

Supplement: Fig. S2 — co-IP viral proteins/IFI6. [file jvi.00760-25-s0002.tif]
