# Supplementary material for: Type I and III interferon responses restrict infection by tick-borne orthoflaviviruses through IFI6
Source: J Virol. 2026 Apr 14;100(5):e00760-25. doi: 10.1128/jvi.00760-25 (PMC13185562; doi:10.1128/jvi.00760-25)
Supplement: Supplemental material — Legends for Fig. S1 and S2; Tables S1 to S3. [file jvi.00760-25-s0003.docx]

**Supplementary material**

**Supplementary figure legends**

**Fig. S1.** Wild-type mice were infected subcutaneously (footpad) or orally with POWV-. At 2-day post-infection, sera were analyzed by ELISA to determine the amounts of IFNα1 secreted. Sera from non-infected mice were used as controls.

**Fig. S2.** **IFI6 does not interact with individual TBEV proteins.** 293T cells were transfected with plasmids expressing IFI6-HA and empty vector (EV) plasmids or plasmids expressing FLAG‐tagged versions of proteins encoded by TBEV. Cells were lysed 24 h post‐transfection, and whole‐cell lysates were immunoblotted with the indicated antibodies (input). The same samples were issued to an immunoprecipitation assay using anti‐FLAG magnetic beads and subsequent staining with the indicated antibodies (FLAG-IP) (n=2).

Table S1. siRNA sequences used for knockdown experiments.

| **siRNA** | **Sequence 5’-3’** |
| --- | --- |
| **IFI6 #1** | GCAGCGUCGUCAUAGGUAA |
| **IFI6 #2** | GCAGAAGGCGGUAUCGCUU |
| **IFI6 #3** | UCGAUAGUGAGGAGGAUGA |
| **IFI6 #4** | CUUCAUGGCCGUCGGAGGA |
| **NT #1** | UGGUUUACAUGUCGACUAA |
| **NT #2** | UGGUUUACAUGUUGUGUGA |
| **NT #3** | UGGUUUACAUGUUUUCUGA |
| **NT #4** | UGGUUUACAUGUUUUCCUA |
| **STAT2 #1** | GGACUGAGUUGCCUGGUUA |
| **STAT2 #2** | GGACUGAGGAUCCAUUAUU |
| **STAT2 #3** | GAGCCCUCCUGGCAAGUUA |
| **STAT2 #4** | GAUUUGCCCUGUGAUCUGA |
| **IFNAR1 #1** | GCGAAAGUCUUCUUGAGAU |
| **IFNAR1 #2** | UGAAACCACUGACUGUAUA |
| **IFNAR1 #3** | GAAAAUUGGUGUCUAUAGU |
| **IFNAR1 #4** | GAAGAUAAGGCAAUAGUGA |
| **IFNLR1 #1** | CACUGGAUCUGAAGUAUGA |
| **IFNLR1 #2** | CCAAGGAGCUGCUAUGUUC |
| **IFNLR1 #3** | CCAGAACCAUCUACACGUU |
| **IFNLR1 #4** | GGCCUGCGCUGGAAGCAUA |
| **KEAP1 #1** | GGACAAACCGCCUUAAUUC |
| **KEAP1 #2** | CAGCAGAACUGUACCUGUU |
| **KEAP1 #3** | GGGAGUACAUCUACAUGCA |
| **KEAP1 #4** | CGAAUGAUCACAGCAAUGA |

Table S2. RT-qPCR primer sequences.

| **Target Gene** | **Forward primer 5’-3’** | **Reverse primer 5’-3’** |
| --- | --- | --- |
| ***GAPDH*** | GGTCGGAGTCAACGGATTTG | ACTCCACGACGTACTCAGCG |
| ***TBEV-Hypr NS5*** | ATCCTGCATTCTGGCACCTC | TACCAAATGGCCCGACTTCC |
| ***POWV-LB NS5*** | GCATGGTCGGATGAACAGAA | GAGCGCTCTTCATCCACCA |
| ***IFNA2*** | Gatggtttcagccttttgga | Gcaagtcaagctgctctgtg |
| ***IFNB*** | Aagcaattgtccagtccca | Tgcattacctgaaggccaag |
| ***IFNL1*** | TCAGCTTGAGTGACTCTTCCAAGG | GCCACATTGGCAGGTTCAAATCTC |
| ***IFNL2/3*** | TCCAGAACCTTCAGCGTCAG | AGGGCCAAAGATGCCTTAGA |
| ***IFNAR1*** | TTCCACATCACAGTATCTACCC | TGCAAATTCCAGCAGAAGCTA |
| ***STAT2*** | GAGCCAGCAACATGAGATTGA | GCCTGGATCTTATATCGGAAGCA |
| ***IFNLR1*** | CCTCCCCAGAATGTGACGC | CCCGCACACTCTTCCACTT |
| ***KEAP1*** | CTGGAGGATCATACCAAGCAGG | GGATACCCTCAATGGACACCAC |
| ***IFI6*** | GTGGAGGCAGGTAAGAAAAAG | ATTCAGGATCGCAGACCAG |

Table S3. sgRNA sequences used for lentiviral vector-mediated CRISPR/Cas9 KO.

| **sgRNA** | **Sequence 5’-3’** |
| --- | --- |
| **IFI6** | CTGCTGCTCTTCACTTGCAG |
| **KEAP1** | AGTACGACTGCGAACAGCGA |
| **NT** | AAGAGCGAATCGATTTCGTG |
